# Temporal and spatial dynamics of scaling-specific features of a gene regulatory network in *Drosophila*

**DOI:** 10.1038/ncomms10031

**Published:** 2015-12-08

**Authors:** Honggang Wu, Manu, Renjie Jiao, Jun Ma

**Affiliations:** 1 Division of Biomedical Informatics, Cincinnati Children's Research Foundation, 3333 Burnet Avenue, Cincinnati, Ohio 45229, USA; 2 State Key Laboratory of Brain and Cognitive Science, Institute of Biophysics, Chinese Academy of Sciences, Datun Road 15, Beijing 100101, China; 3 University of Chinese Academy of Sciences, Beijing 100080, China; 4 Department of Biology, University of North Dakota, 10 Cornell Street Stop 9019, Grand Forks, North Dakota 58202, USA; 5 Sino-French Hoffmann Institute, Guangzhou Medical University, 195 Dongfengxi Road, Guangzhou 510182, China; 6 Division of Developmental Biology, Cincinnati Children's Research Foundation, 3333 Burnet Avenue, Cincinnati, Ohio 45229, USA

## Abstract

A widely appreciated aspect of developmental robustness is pattern formation in proportion to size. But how such scaling features emerge dynamically remains poorly understood. Here we generate a data set of the expression profiles of six gap genes in *Drosophila melanogaster* embryos that differ significantly in size. Expression patterns exhibit size-dependent dynamics both spatially and temporally. We uncover a dynamic emergence of under-scaling in the posterior, accompanied by reduced expression levels of gap genes near the middle of large embryos. Simulation results show that a size-dependent Bicoid gradient input can lead to reduced *Krüppel* expression that can have long-range and dynamic effects on gap gene expression in the posterior. Thus, for emergence of scaled patterns, the entire embryo may be viewed as a single unified dynamic system where maternally derived size-dependent information interpreted locally can be propagated in space and time as governed by the dynamics of a gene regulatory network.

## Abstract (web summary)

How pattern formation is regulated relative to the size of an organism is unclear. Here, Wu *et al.* take data from gap gene expression in flies of different sizes together with simulations, identifying how scaling emerges dynamically and that local patterning influences global gene regulatory networks.

## Graphical abstract

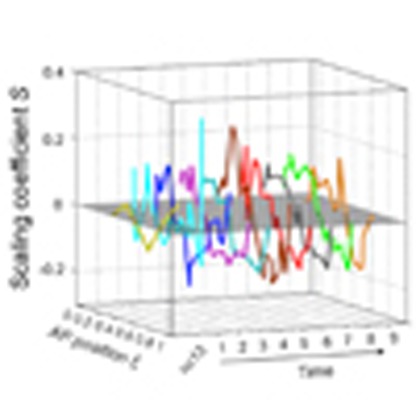

Formation of patterns that are generally proportional to an individual's body size is an intriguing feature of animal development^1^,^2^,^3^,^4^. There are two aspects directly relevant to this developmental scaling problem—scaled tissue specification and coordinated tissue expansion^5^,^6^. In many developmental systems, the events controlling these two aspects are connected both temporally and mechanistically^7^,^8^,^9^,^10^, and recent studies have investigated mechanisms controlling the formation and action of morphogen gradients whose profiles are scaled with the expanding size of a tissue^11^,^12^,^13^,^14^. In other developmental systems, tissue specification and tissue expansion can take place in a temporally sequential manner^6^. For example, formation of scaled patterns in the chick and mouse neural tube is controlled in two sequential phases, morphogen-induced progenitor specification followed by cell-type-specific growth^15^. In *Drosophila* embryos, patterning along the anterior-posterior (AP) axis can be viewed purely as a patterning problem because the physical size of the patterning system, the embryo, is predetermined at an earlier stage of the life cycle (that is, oogenesis) and does not change during the time of pattern formation^6^,^16^. This system thus provides a unique window to probing how scaled patterns emerge from the dynamic operation of a gene regulatory network without the entanglement of growth.

Gap genes are situated at the top of the zygotic regulatory hierarchy for instructing the AP patterning outcome in *Drosophila*^17^,^18^,^19^,^20^,^21^,^22^,^23^,^24^. These genes respond initially to maternal inputs such as the Bicoid (Bcd) morphogen gradient and, subsequently, rely on extensive cross-regulatory mechanisms that form an intricately wired regulatory network^19^,^25^,^26^,^27^. Previous studies have generated useful insights into the regulatory mechanisms controlling the spatial and temporal dynamics of gap gene expression^25^,^28^,^29^,^30^. But how these dynamics are related to, and impacted by, embryo size remains poorly understood. In addition, while the relationship between the scaling properties of the Bcd gradient input and the expression of the gap gene *hunchback* (*hb*) has been studied quantitatively^6^,^31^, whether (and if so how) this maternal input may impact the dynamic operation of the entire gap gene network in relation to embryo size remains to be resolved.

The concept of gene regulatory networks provides a mechanistic view of how the precise and reproducible patterning and cell fate specification are controlled^24^. In addition to the scaling problem in embryonic pattern formation that we investigate in the current study, developmental robustness can also be understood in the context of other types of variability that a developmental system must face, such as molecular noise^32^,^33^,^34^,^35^, environmental fluctuations^18^,^36^,^37^ and genetic variations^38^,^39^. While the term robustness (also often referred to as canalization) projects an effective image of developmental systems producing reliable outcomes in spite of the various uncertainties, robustness may not be viewed as an absolute concept. There is a ‘sensitive' side of developmental systems that is exemplified by concentration-dependent actions of regulatory proteins^24^,^31^,^32^,^40^,^41^ or human diseases caused by either having an extra copy or mutating a single copy of a regulatory gene^42^,^43^. Thus experimental and theoretical studies that probe molecular origins and limits of robustness in well-defined systems can lead to fundamental insights into how developmental programs are controlled. With respect to the scaling problem in *Drosophila* AP patterning that we investigate here, accumulating evidence suggests that scaling is imperfect^6^,^38^,^39^,^44^,^45^, but the underlying molecular mechanisms are not fully resolved, particularly for the posterior part of the embryo^6^. Since gap genes are the earliest responders to maternal inputs and form a well-characterized regulatory network, systematically evaluating their expression properties in relation to embryo size will likely lead to new insights into how size-dependent (that is, scaling-specific) features of a patterning system emerge. Such new insights can strengthen our knowledge of the concept of developmental robustness in terms of its underlying molecular origins and limits.

Here we perform quantitative studies in *Drosophila* embryos that differ significantly in egg length and permit reliable extraction of scaling-specific information. We quantify the expression properties of six gap genes, *orthodenticle* (*otd*), *hb*, *giant* (*gt*), *Krüppel* (*Kr*), *knirps* (*kni*) and *tailless* (*tll*) in these embryos. Our results reveal that scaling-specific features of gap gene expression are highly dynamic in both space and time. We show that gap expression boundaries in the anterior exhibit a general over-scaling (over-scaling is defined as an excessive compensation for embryo length to give rise to a more posteriorly located boundary in large embryos relative to small embryos), which is consistent with the scaling properties of the Bcd gradient input^6^,^31^,^46^. The expression boundaries in the posterior trunk region of the embryo undergo a spatially and temporally concerted dynamic under-scaling (under-scaling is defined as an insufficient compensation for embryo length to give rise to a more anteriorly located boundary in large embryos relative to small embryos). We perform simulation studies to probe the origins and impacts of an experimentally detected divergence in gap gene expression levels between large and small embryos. Our results support a view that the divergence in *Kr* expression level is a ‘localized' scaling-specific feature that can originate from the size-dependent properties of the Bcd gradient and exert long-range and dynamic effects on gap gene expression behaviour in the posterior. Our study suggests that the molecular interactions (of the gap gene network) that control gene expression behaviour within individual cells (nuclei) also mediate the propagation of size-dependent ‘information' in space and time throughout the embryo. Thus, the entire embryo may be viewed as a single unified dynamic system, in which a ‘localized' interpretation of a size-dependent morphogen input can impact the dynamics and outcome of the patterning landscape as a whole. Such a ‘holistic' view may be of general importance to studies of problems in developmental biology.

## Results

### Experimental design and data set

To facilitate experimental investigation of scaling-specific properties of AP patterning, we took advantage of two *Drosophila* inbred lines that had been artificially selected to lay large and small eggs^39^,^46^ (hereafter referred to as the large-egg and small-egg line, respectively). Embryos from these two lines are referred to as the large and small embryos, respectively. Their mean lengths, 482.0±20.9 μm (mean±s.d.) and 408.6±16.8 μm, differ by ∼15% (*P*=2.94 × 10^−26^; length measurements are from T3 embryos for *hb* FISH (fluorescence *in situ* hybridization); see Supplementary Table 1 for *n* and below for further details). We used whole mount mRNA FISH to analyse the expression patterns of six gap genes that form boundaries distributed across the entire length of the embryo. Our experimental and imaging procedures were designed to avoid any nonlinear steps of signal amplification or adjustments. Under our experimental and analytical framework^41^, the extracted intensities of the fluorescent signals preserve a linear relationship with the cytoplasmic mRNA concentrations (in arbitrary units, a.u.). To permit direct comparisons, both sets of embryos were treated side-by-side at both experimental and imaging steps.

To facilitate the reconstruction of the temporal evolution of gene expression patterns, we sorted embryos into well-resolved temporal classes using morphological markers. This study focuses primarily on nuclear cycles (nc) 13 and 14, during which gap gene transcripts have begun to accumulate to levels suitable for reliable quantitative measurements. This is also the time during which gap genes have begun to cross-regulate among themselves in an intensifying way^17^. For embryos at nc14, we divided them into nine time classes in a manner to ensure that each time class had a sufficient sample size for each and every gene analysed (Supplementary Table 1; see Methods for estimated time durations). In total, the data set generated in this study consists of data from 2,202 embryos, with 20 expression boundaries spanning across the entire AP length and across 10 time internals. Our data set permitted us to extract quantitative information about gap gene expression with regards to (1) their spatial features (that is, expression domains and boundary positions at a given time), (2) their temporal dynamics (that is, movements of domain boundaries and threshold-crossing positions as a function of time) and (3) their scaling-specific features (that is, relationships between the above mentioned properties and embryo size).

### Spatial registry and movements of gap expression boundaries

**Figure 1:**
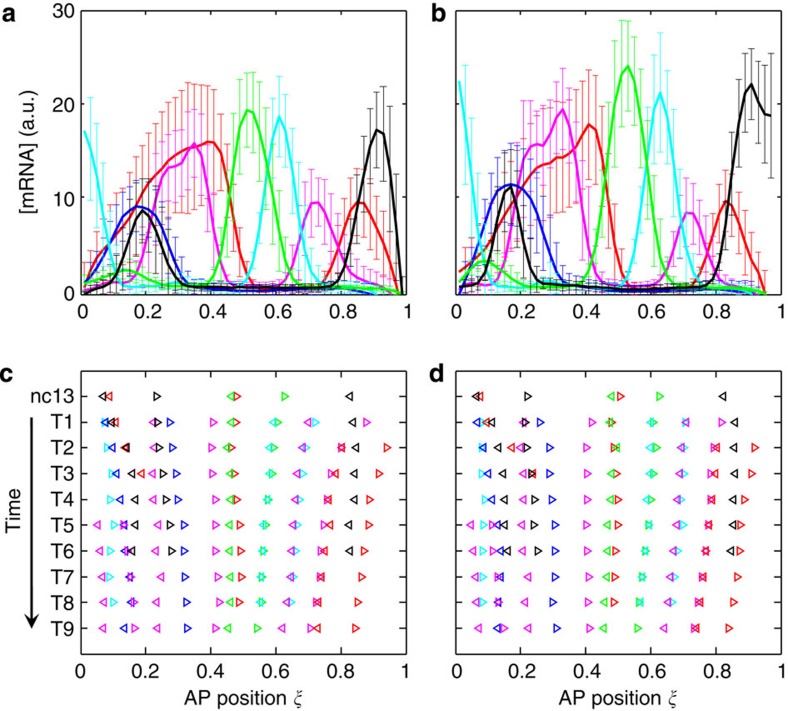
Spatial registry and positional dynamics of gap gene expression boundaries. (**a**,**b**) Shown are expression profiles of the indicated six gap genes in large (**a**) or small (**b**) embryos at the peak time (in expression) of nc14 (T2 and T3 combined) as a function of relative AP position *ξ*=*x*/*L* (0 and 1 denote anterior and posterior, respectively). For each gene, the intensity data extracted from an embryo along the AP axis were background-subtracted without any further adjustments. The mean and s.d. (shown as error bars) of the intensity data of the indicated genes are plotted for each of the two groups of embryos. (**c**,**d**) Shown are mean positions of gap expression boundaries in large (**c**) or small (**d**) embryos at the indicated time classes. Anterior and posterior boundaries are shown as left- and right-pointing triangles, respectively. Note that not all boundaries are present at all times (see Methods). Colour code from a to d: red, *hb*; magenta, *gt*; green, *Kr*; cyan, *kni*; blue, *otd*; black, *tll*. See Supplementary Table 1 for n.

Figure 1a,b shows superimposed profiles of gap gene expression as a function of relative AP position in large and small embryos, respectively. Throughout this report, relative positions along the AP axis are expressed as values of fractional embryo length, *ξ*=*x*/*L*, with 0 denoting the anterior pole and 1 the posterior pole. Our results shown in Fig. 1a,b yield the following spatial registry of expression boundaries for both large and small embryos along the AP axis: *kni*1>*otd*1>*tll*1>*hb*1/*gt*3>*tll*2>*otd*2>*gt*4>*hb*2/*Kr*1>*Kr*2/*kni*2>*kni*3/*gt*5>*gt*6/*hb*3>*tll*3>*hb*4 (see Supplementary Fig. 1 for boundary nomenclature). Two additional boundaries, *gt*1 and *gt*2, which emerge at later times in nc14, can be included in this spatial registry that would remain identical between the two sets of embryos (Supplementary Fig. 1). These results show that, despite the considerable difference between their average lengths, embryos from the two inbred lines have an identical spatial registry of the expression boundaries of gap genes. They indicate that, at this initial level of analysis and consistent with the well-acknowledged framework of developmental robustness, expression patterns of gap genes are generally robust to variations in embryo lengths.

To analyse quantitatively the expression boundaries of gap genes and characterize their temporal movements during development, we measured their relative positions in individual embryos (Fig. 1; Methods). Figure 1c,d shows the mean positions of each of the analysed boundaries in the large and small embryos, respectively, at each of the time classes (see Supplementary Fig. 2 for s.d.). These results provide a visual representation summarizing how the gap expression boundaries move along the AP axis as a function of time. We can see readily that the boundaries located in the anterior and posterior parts of the embryos tend to exhibit movements toward mid-embryo (for example, *otd*2 and *hb*3). This is in contrast to boundaries near mid-embryo (for example, *gt*4, *hb*2 and *Kr*1) or near the anterior cap (*kni*1), whose positions remain relatively stable as a function of time. The overall dynamic movements of gap expression boundaries in embryos from the two inbred lines are generally similar to those in other characterized wild-type strains^28^,^29^,^30^.

### Location-dependent scaling of gap expression boundaries

To evaluate AP patterning properties in relation to embryo length, we compared directly the positions of gap expression boundaries in large and small embryos. Our analysis reveals measurable differences between large and small embryos in either the boundary positions at a given time or their dynamic movements over time (Supplementary Fig. 2, and see legend for discussions of specific examples). In addition, the moving span of a boundary (that is, the shift of a boundary's relative AP position during a period of time) can also differ between large and small embryos (Supplementary Fig. 2 legend).

To quantify the relationship between AP patterning properties and embryo size, we employed a parameter, the scaling coefficient *S*, which was measured by pooling large and small embryos for a given time class (Methods; Supplementary Fig. 3). Under our current definition^6^, *S*=0 denotes perfect scaling, that is, a boundary position (expressed as a relative AP position *ξ*) exhibits a sample-wide insensitivity to variations in embryo length^44^. When *S*<0 or *S*>0, the boundary is under- or over-scaled, respectively, indicating that an increase in embryo length is either inadequately (under) or excessively (overly) compensated at the boundary position of a gene expression domain. Therefore, *S* quantifies exclusively boundary position variations derived from imperfect scaling despite the existence of other inevitable sources of noise that can also contribute to boundary variations (see Supplementary Fig. 4 for results supporting the conclusion that scaling contributes to the overall precision of gap expression boundaries).

**Figure 2:**
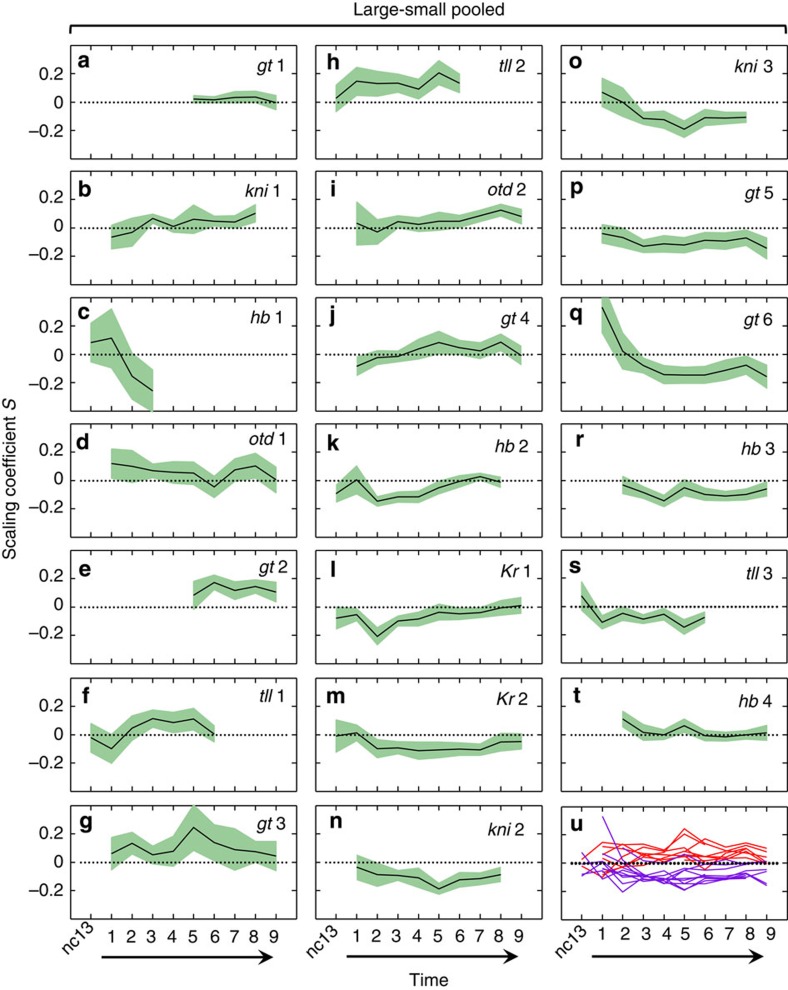
Temporal evolution of scaling coefficient of gap expression boundaries. (**a**–**t**) Shown are scaling coefficient *S* profiles for individual gap expression boundaries at the indicated times. Panels are ranked according to the AP registry of the boundaries (top left most anterior, bottom right most posterior). A dotted line denotes *S*=0 and shaded bands are 95% CI of the linear regression slope (Supplementary Fig. 3). (**u**) Superimposed plot of *S* profiles for all the boundaries from **a** to **t** (except the fast-decaying *hb*1), with two colours denoting boundaries located in the anterior (dark red) or the posterior trunk region (purple).

Figure 2a–t shows the *S* values for individual expression boundaries plotted as a function of time, with the 95% confidence intervals (CI) shown as shaded bands. These profiles reveal diverging scaling properties in the anterior and posterior parts of the embryo. In particular, while the gene expression boundaries in the anterior have a tendency of being over-scaled (that is, *S*>0), those in the posterior tend to be under-scaled (that is, *S*<0). In addition, expression boundaries located near the middle of the embryo (for example, *gt*4) or close to either pole (for example, *gt*1, *hb*4) have a tendency of exhibiting a nearly perfect scaling, particularly at later times of nc14. To provide a visual representation of these scaling trends, we generated an illustrative, superimposed plot of the *S* profiles for gap expression boundaries (Fig. 2u). Here the over- and under-scaled boundaries become readily perceivable. These results show that gap expression boundaries located in different parts of the embryo have different scaling-specific properties, that is, scaling is not a spatially uniform feature of the gap gene network.

### Spatially and temporally concerted posterior under-scaling

**Figure 3:**
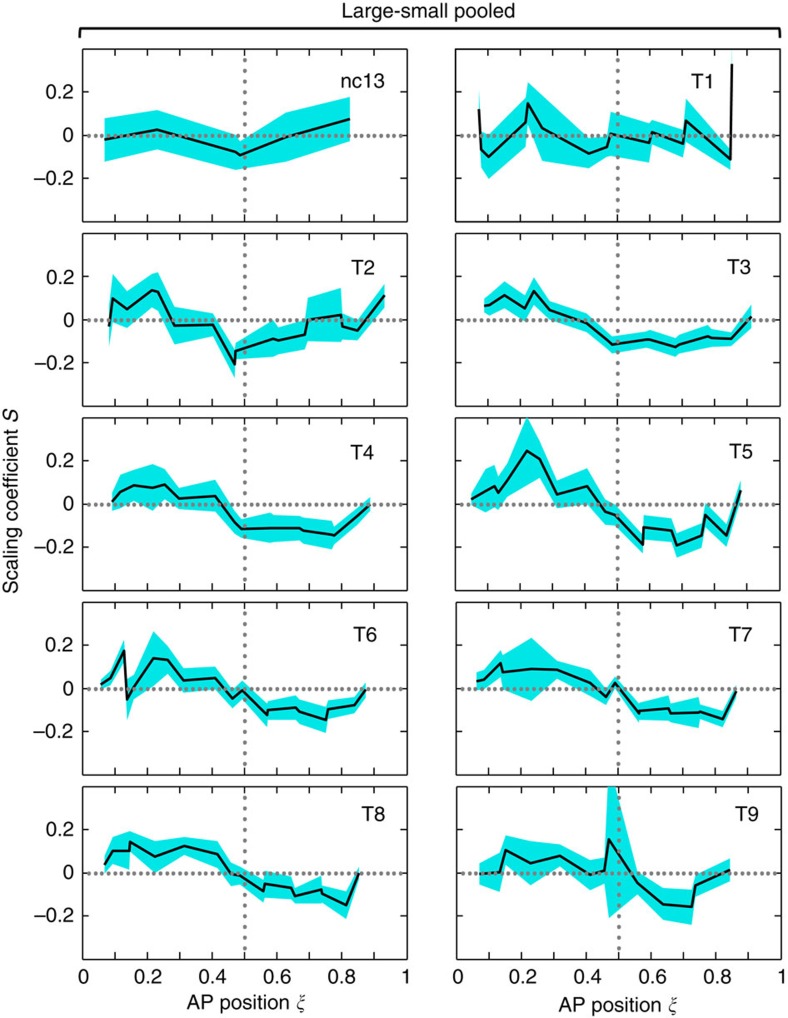
Scaling coefficient profiles as a function of AP position. Shown are measured *S* profiles of gap expression boundaries plotted as a function of AP position at each of the 10 time classes, with blue bands representing 95% CI of *S*. The two dotted lines denote mid-embryo (relative AP position *ξ*=0.5) and perfect scaling (*S*=0), respectively.

A noted feature of the results shown in Fig. 2 is that, for expression boundaries in the posterior trunk region of the embryo (for example, *kni*2, *kni*3 and *gt*6), the degree of under-scaling is the greatest around mid-nc14 (that is, for *kni*3 at T5, *S*=−0.19±0.06; error bar is 95% CI). At earlier times, many of these boundaries are not under-scaled, with some even exhibiting over-scaling. An example is *gt*6, which has the highest degree of over-scaling at T1 (*S*=0.33±0.18). But this over-scaling vanishes precipitously (*S*=0.02±0.13 at T2) to become under-scaled as embryos progress further into nc14 (for example, *S*=−0.14±0.07 at T4). Figure 3 shows *S* profiles for expression boundaries detected along the AP axis at individual time classes (see Supplementary Fig. 5 for a three-dimensional illustration). Here, under-scaling of gap expression boundaries in the posterior trunk region of the embryo at mid-to-late times of nc14 exhibits itself as one of the most visible features. This feature can be further appreciated when evaluated against the *S* profiles at earlier times (nc13, T1 and T2) before the emergence of under-scaling for these boundaries.

Another feature displayed in Fig. 3 is a ‘wave-like' under-scaling that emerges near mid-embryo and propagates toward the posterior. Specifically, a sudden dip (*S* amplitude trough) develops around middle embryo at T2. This *S* amplitude trough moves towards the posterior to cause a broader swath of the embryo become under-scaled. By the time of T6, the *S* amplitude trough has reached a location near *ξ*=∼0.8, while at the same time the region near mid-embryo has been fully recovered. The *S* amplitude trough never reaches the posterior pole region marked by the *hb*4 boundary (see also Fig. 2t), and portions of the posterior trunk region never recover fully. Together, our analyses of the *S* profiles show that the scaling-specific features of the AP patterning network emerge dynamically with regard to both space and time.

### Large embryos have reduced levels of gap expression in the middle

**Figure 4:**
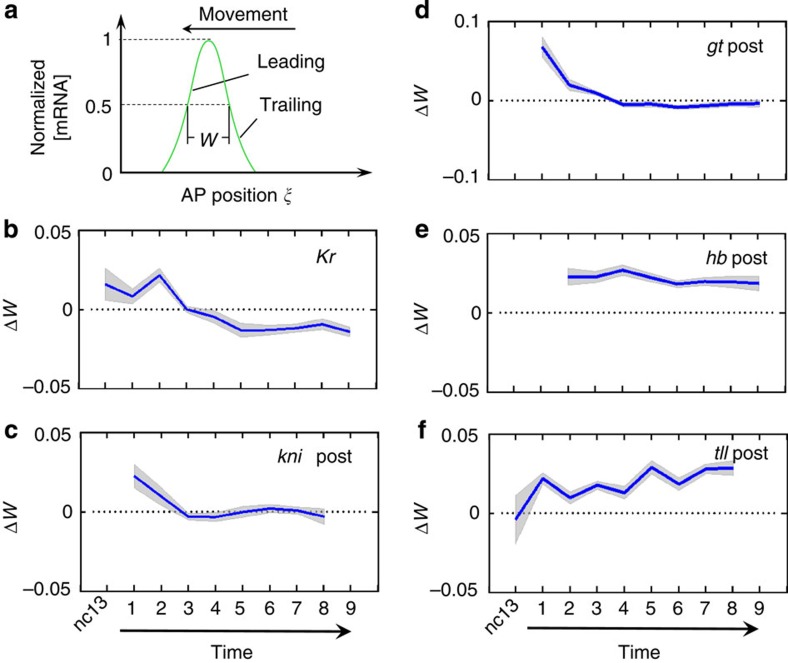
Dynamic changes in gap expression domain widths. (**a**) Shown is a schematic diagram of an expression domain illustrating the definition of its width (*W*), the direction of its movement during nc14, and a depiction of its leading and trailing boundaries. (**b**–**f**) Shown are differences in domain widths (expressed as values relative to embryo length) between large and small embryos as a function of time. Shaded band for each panel represents s.d. of Δ*W*.

The location- and time-dependent scaling properties of gap expression boundaries described above represent new discoveries that have not been documented previously. They suggest that, despite extensive studies, our current knowledge about how this regulatory network operates remains incomplete. To probe potential mechanisms, we gauged the effects of embryo size on spatiotemporal patterning by analysing the alterations to gap expression domain widths in time (see Fig. 4a for a cartoon depicting a moving expression domain that has a ‘leading' boundary and a ‘trailing' boundary in a shifting landscape). Here we measured the widths of each expression domain (expressed as values relative to embryo length) in the large and small embryos to obtain a difference, Δ*W* (Methods; Supplementary Fig. 6). Figure 4b–d shows that Δ*W* for *Kr*, *kni* and *gt* domains in the posterior trunk region declines as a function of time, suggesting that these domains experience a greater degree of contraction in large embryos relative to small embryos. This effect for the *Kr* domain is most striking, where an initial positive Δ*W* becomes a negative value at later times of nc14 (Fig. 4b). Figure 4e,f shows that, for expression domains closer to the posterior pole (that is, the posterior *hb* and *tll* domains), Δ*W* remains generally a positive value throughout nc14 (Supplementary Fig. 6).

**Figure 5:**
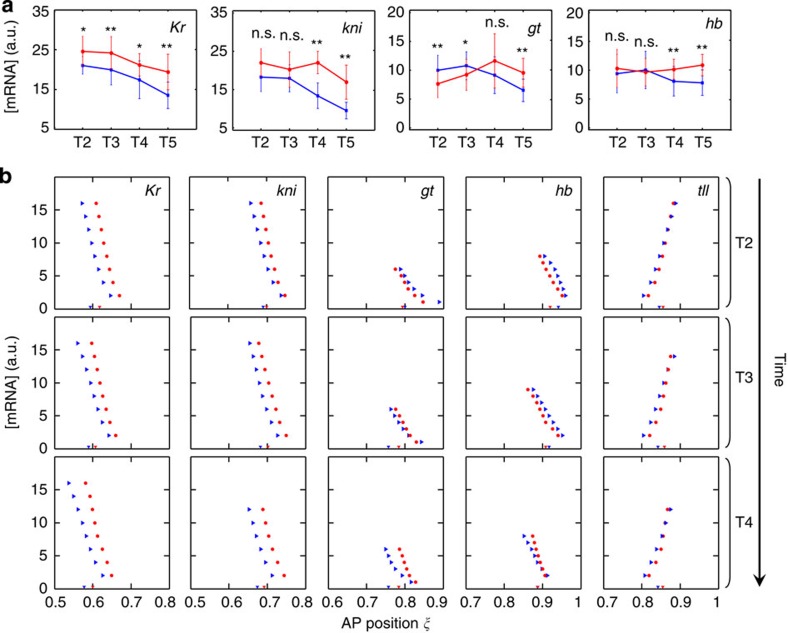
Large embryos exhibit weaker or weakening gap expression levels in the posterior. (**a**) Gap gene expression levels in the posterior trunk region at T2–T5. For each embryo, the expression level is estimated as the mean across the three highest intensity bins of a domain. Blue and red denote large and small embryos, respectively. Error bars are s.d. Results of Student's *t*-tests are given, with n.s., * and ** denoting not significant, *P*<0.05 and *P*<0.01, respectively. (**b**) Shown are AP positions at which a mean intensity profile crosses given thresholds. The trailing boundaries for *Kr*, *kni*, *gt* and *hb* as well as the leading boundary of posterior *tll* domain are shown. Measured boundary positions of the large and small embryos are indicated at the bottom of each panel for reference. Blue, large embryos; red, small embryos. See Supplementary Table 1 for n.

To further probe potential mechanisms or events associated with the emergence of under-scaling in the posterior trunk region, we compared the expression levels of gap domains in large and small embryos (see Methods). Figure 5a shows that, relative to small embryos, large embryos exhibit properties of either decreased or progressively decreasing expression levels in the posterior trunk region. In particular, throughout nc14, *Kr* level is persistently lower in large embryos than in small embryos (Fig. 5a; Supplementary Fig. 1). While the expression levels of both the *kni* and *hb* domains at earlier times of nc14 are comparable between large and small embryos, their levels in large embryos progressively become lower relative to small embryos (Fig. 5a). The posterior *gt* domain in large embryos exhibits a particularly dynamic decrease in expression output, with a higher level at earlier times but a lower level at later times (Fig. 5a). The fact that large embryos do not have universally (gene- and time-wise) low expression levels also rules out systematic experimental errors as the origin of differential expression levels. Together, the dynamic differences in gap domain widths (Fig. 4) and expression levels (Fig. 5a) suggest that, relative to small embryos, large embryos tend to have a weaker gene expression output particularly near the middle at the *Kr* domain.

### Evaluating potential originators of posterior under-scaling

To further evaluate which part of the embryo may play an originator role in the emergence of posterior under-scaling, we compared the positions at which a mean expression profile crosses given thresholds in large and small embryos. Unlike measurements of boundary position or domain width, which utilize normalized intensity data (Fig. 4a; Methods), threshold-crossing analysis was based on unnormalized data, thus also taking into account the expression level of a gene at a time. Figure 5b shows scatter plots of threshold-crossing positions of the trailing boundaries in the posterior trunk region as well as the leading *tll*3 boundary (Supplementary Fig. 7). Here we focus on time classes T2–T4, during which the observed under-scaling begins to emerge (Figs 2 and 3; Supplementary Fig. 5).

Our analysis of threshold-crossing positions of gap expression profiles leads to the following findings. First, when differences between large and small embryos are considered, the trailing *Kr* boundary (that is, the posterior boundary of the central *Kr* domain) moves in a manner that is earliest in time and largest in moving span (see above for definition) among all the trailing boundaries in the posterior trunk region (Fig. 5b). Second, the threshold-crossing positional differences (between large and small embryos) for leading boundaries are generally less than trailing boundaries (Supplementary Fig. 7). Third and importantly, the threshold-crossing positions of the *tll*3 boundary do not expand appreciably more from the posterior pole in large embryos than in small embryos (Fig. 5b). These results argue against the posterior pole as the originator of under-scaling.

### Long-range and dynamic impacts of a lowered *Kr* level

Our results described thus far suggest that the size-dependent divergence in gap expression levels near mid-embryo (Fig. 5a) is itself a scaling-specific feature that could potentially account for some of the observed patterning behaviour in the posterior trunk region. To directly evaluate this possibility, we performed simulation studies based on a gene circuit model of the gap gene network that correctly simulates dynamic domain boundary shifts^25^ as well as the effects of embryo-to-embryo Bcd variation^19^,^47^. Here we specifically reduced the maximal synthesis rates of gap proteins without any other adjustments to the model^14^ (Methods). In essence, our analysis was specifically designed to simulate how a reduction in gap protein levels—as a given—affects the behaviour of the operation of the gap gene network. In our analysis, we reduced the maximal synthesis rates of one protein at a time (Kr, Kni, Gt or Hb) or reduced all four simultaneously.

Our results are shown in Supplementary Fig. 8a–e, where protein expression profiles of different gap genes are colour-coded and plotted as a function of relative AP position. They reveal the following findings. First, individually reducing the synthesis rate of Hb or Gt in the embryo only had modest and locally restricted effects on gap gene expression patterns in the posterior (Supplementary Fig. 8a,b). Second, simultaneously reducing the synthesis rates of all four proteins did not cause a strong alteration in expression boundaries (Supplementary Fig. 8c). Third and importantly, individually reducing synthesis rate of either Kr or Kni led to long-range effects on gene expression boundaries in the posterior (Supplementary Fig. 8d,g). However, unlike Kr level reduction that could recapitulate qualitatively the anterior shifts of several expression boundaries (for example, Gt5, Gt6 and Hb3), reducing Kni level caused them to shift posteriorly (compare panels e and h in Supplementary Fig. 8).

Reducing Kr level on its own can dynamically affect the expression levels of other gap proteins in the posterior. While the Kni expression level is lowered throughout nc14, Gt expression is higher at earlier times only to become lower at later times (Supplementary Fig. 8f). This simulated dynamic property is qualitatively very similar to the experimentally observed mRNA expression behaviour of these two genes in the posterior trunk region (Fig. 5a). Furthermore, reducing the synthesis rate of Kr can lower the Kni level but not the other way around (Supplementary Fig. 8f,i). Together, these results suggest that the experimentally observed *Kr* mRNA level divergence between large and small embryos is a scaling-specific feature that on its own can exert long-range and dynamic effects on gap gene expression behaviour in the posterior (Supplementary Fig. 8 legend).

### Bcd input properties can impact gap gene network through *Kr*

The long-range effects of Kr protein level reduction documented by our simulation studies raised an important mechanistic question. If the experimentally observed divergence in *Kr* mRNA level (Fig. 5a) was indeed responsible, at least partially, for some of the scaling-specific features in the posterior trunk region, what properties might have led to such a divergence? To address this question, we turned our attention to Bcd (refs 6, 31). One important property of the Bcd gradient is that the large-embryo profile intersects with the small-embryo profile at a critical position^6^, such that large embryos have higher concentrations anterior to the critical position and lower concentrations posterior to it^3^,^46^. The critical position lies anterior to the *Kr* domain, so that large embryos have lower Bcd concentrations at the location of *Kr* expression than small embryos^46^. Bcd is known to activate *Kr* reporter constructs^48^,^49^, a result supported by *in silico* reconstructions of the gap gene network from expression data^19^,^25^,^50^. A positive role for Bcd in *Kr* transcription suggests that Bcd gradient properties may contribute to (or be responsible for) the experimentally observed *Kr* mRNA level divergence between large and small embryos. To directly test this possibility, we performed simulation studies where we adjusted the amplitude and the relative length scale of the Bcd gradient in ways that are consistent with experimentally observed gradient properties in large embryos. In essence, we compared two otherwise identical embryo systems except for a difference in the Bcd gradient input that is designed to mimic a difference in embryo size.

**Figure 6:**
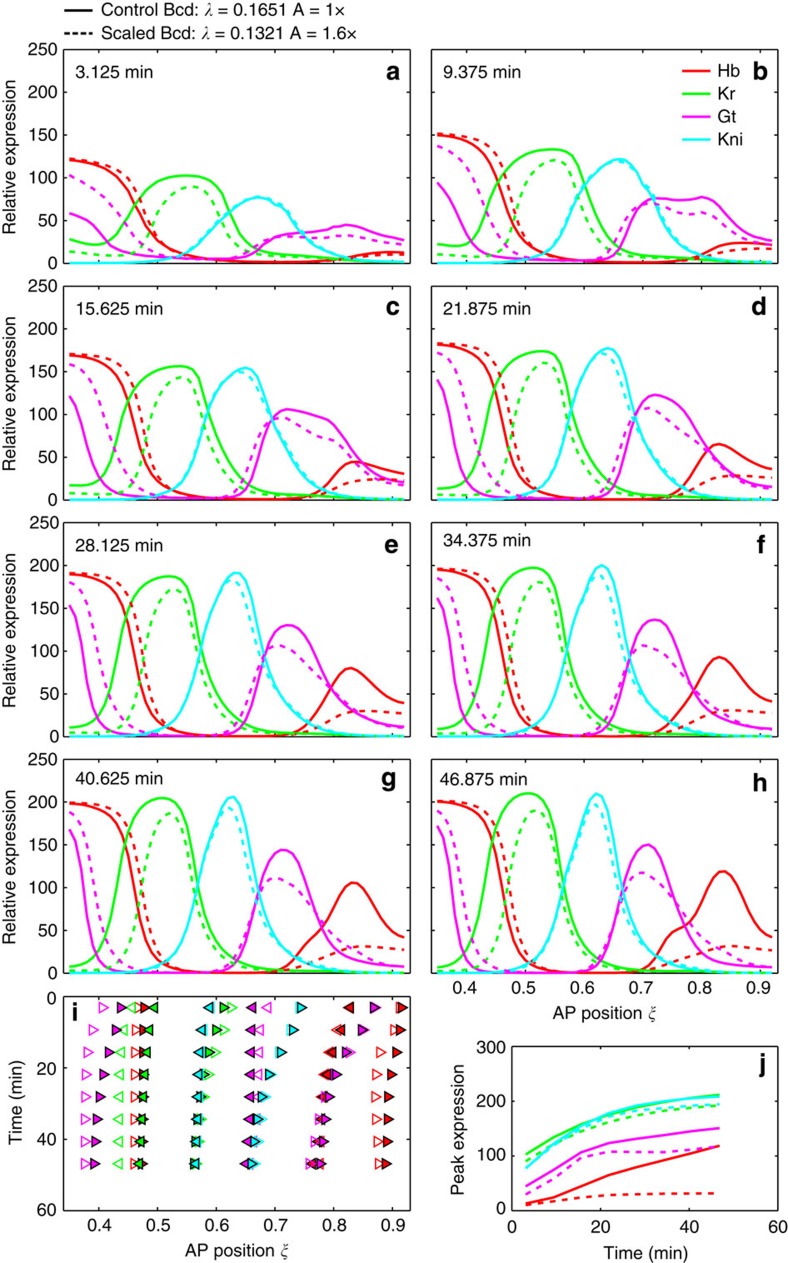
Simulation of Bcd scaling in the gene circuit model of gap genes. (**a**–**h**) Model output for Hb, Kr, Gt and Kni during cleavage cycle 14, at 3.125 (early T1), 9.375 (late T1), 15.625 (early T2), 21.875 (late T2), 28.125 (T3), 34.375 (T4), 40.625 (T6) and 46.875 (T8) minutes after the thirteenth nuclear division. Simulations using control^19^ or scaled Bcd are shown as solid or dashed lines, respectively. The scaling of Bcd in large embryos was based on data from Cheung *et al.*^46^ (see Methods for details). The control Bcd profile was the median profile from wild-type embryos used by Manu *et al.*^19^ to fit the model. The invariance of the Bcd length scale in absolute units was represented by reducing the length scale by 20%—from 0.1651 EL to 0.1321 EL—in the relative position units utilized in the model. Furthermore, a stronger Bcd source, caused by the increased amount of *bcd* mRNA in large embryos^46^, was modelled by multiplying the source strength (*A*), and hence Bcd concentration, by 1.6. These values of Bcd amplitude and length scale imply a critical position at *ξ*=0.311 (see Supplementary Fig. 9 for simulation experiments of adjusting the critical position). Control and scaled simulations are indicated in the legend above **a**. (**i**) Time series of boundary positions in the model output; see above for time points. Anterior and posterior boundaries are represented by left- and right-pointing triangles, respectively. The colour key is shown in **b**. Control and scaled Bcd simulations are shown as open and filled triangles, respectively. (**j**) Time series of the expression level (in a.u.) at the peak of the posterior Hb, central Kr, posterior Gt and abdominal Kni domains. Control and scaled simulations are shown as solid and dashed lines, respectively. Kr2, Kni2, Kni3 and Gt5 boundaries shift to the anterior. The reduction in Kr expression at the domain peak is relatively constant in time, whereas the difference between control and scaled Bcd simulations for Gt and Kni increases in time.

Our simulation results (Fig. 6; Supplementary Fig. 9) show that the scaling properties of the Bcd input can cause Kr level reduction in the simulated large embryos. Importantly, as we moved the critical position further anterior and away from the Kr expression domain, Kr level became progressively lower in the simulated large embryos (Supplementary Fig. 9). This documents a causal effect. In addition, the impact of the scaling properties of the Bcd gradient is not restricted to Kr expression level in the simulated large embryos. Other gap proteins in the posterior trunk region also exhibit reduced levels (Fig. 6j; Supplementary Fig. 9c,f,i). Importantly, while Kr has a lower level in the simulated large embryos throughout nc14, other gap proteins (most notably Kni and Gt) exhibit dynamic weakening in a manner that resembles the experimentally observed behaviour (Fig. 5a).

The expression boundaries of gap genes in the posterior trunk region of the simulated large embryos also exhibit differences relative to their small counterparts in their spatiotemporal dynamics. The properties of two boundaries, Kni2 and Gt5, are particularly noteworthy. These two boundaries in large embryos exhibit an anterior shift relative to small embryos (that is, they are under-scaled) at early times, but such an under-scaling becomes ‘recovered' at later times. These simulated dynamic properties are reminiscent of the ‘wave-like' initiation and recovery of under-scaling observed experimentally (Fig. 3; Supplementary Fig. 4). Together these simulation studies provide important mechanistic insights into how the regulatory logic of the gap gene network can permit a ‘localized' scaling-specific feature, namely *Kr* mRNA level divergence near mid-embryo, to exert its long-range effects dynamically. In addition, under the framework of the gene circuit model^19^, this ‘localized' scaling-specific feature near mid-embryo can originate from the scaling properties of the Bcd gradient.

## Discussion

Anterior-posterior patterning in *Drosophila* embryos represents an excellent experimental paradigm for studying regulatory network operation and developmental robustness. Despite extensive studies, it is poorly understood how the AP patterning network adapts its operation to the size of an embryo in achieving developmental robustness. Here we perform experimental and simulation studies to gain mechanistic insights into this question. We use two inbred lines that together offer an enhanced spread in embryo length within a species to allow an efficient extraction of scaling-specific features of gap expression properties. We show that scaling is neither absolute in space nor constant in time. We uncover a spatially and temporally concerted dynamic emergence of under-scaling of gap expression boundaries in the posterior trunk region, in general agreement with *even-skipped* (*eve*) expression behaviour in this part of the embryo^6^. An important design feature of our experimental data set, which is the first of its kind, is to permit direct comparisons of gap expression levels in relation to embryo size. Our experimental and simulation results support a view that the dynamic emergence of under-scaling in the posterior trunk originates ‘locally' as a result of lowered expression levels of gap genes near the middle of large embryos.

Our results show that gap expression boundaries located in the anterior are generally over-scaled (Fig. 2), which is consistent with the scaling properties of the Bcd gradient input in this part of the embryo^6^,^46^. In the anterior, the *tll*2 and *otd*2 boundaries are also sensitive to input from the terminal system^51^,^52^. These boundaries exhibit posterior movements (Fig. 1c,d; Supplementary Fig. 2) that coincide with an intensification of over-scaling as embryos progress into nc14 (Fig. 2). These results are consistent with the suggestion that Bcd and terminal inputs interact to specify patterning properties in the anterior of the embryo^21^,^22^,^23^. But the scaling properties of the Bcd gradient are inadequate to explain the scaling-specific features in the posterior^6^. It thus remained an open question as to how such features might emerge dynamically and how they might be controlled. The extensive interactions between gap genes in the posterior trunk region^19^,^25^,^26^,^53^,^54^,^55^ suggest that regulatory networks and/or maternal inputs other than Bcd may play critical roles in patterning this part of the embryo. But our observed dynamic emergence of under-scaling is a new finding and, thus, a fresh conceptual framework is required to gain mechanistic insights. Our simulation results suggest that the scaling properties of the Bcd gradient input can lead to the experimentally observed *Kr* mRNA level divergence between large and small embryos. Thus long-range propagation of ‘localized' effects may represent an important and general mechanism in dynamically guiding the emergence of size-dependent features of patterning systems.

Our current simulation studies were not designed to systematically and quantitatively fit all parameters to experimental data, an analysis that will require future efforts and data. In addition, all of the parameters used in our current simulation studies had been derived from fitting to training data sets that did not have the enhanced spread in embryo size. Naturally our simulated results are not expected to match perfectly with experimental observations for all the individual aspects even on a qualitative basis. In simulated large embryos, both the Hb2 and Kr1 boundaries shift posteriorly (Fig. 6a–h), whereas either an anterior shift or no significant shift is observed at early and later times of nc14, respectively, in the experimental data (Fig. 2k,l; Supplementary Fig. 2). The posterior Hb domain is expressed at lower levels in simulations compared with experimental data. Both of these disagreements with data occur at the edges of the modelled region, and might stem from regulators omitted in the model—the head gap genes and *huckebein* in the anterior and posterior, respectively. In addition, both empirical^56^,^57^,^58^ and recent modelling studies^59^,^60^ suggest a complex regulatory relationship between Hb and Kr in forming the Hb2 and Kr1 boundaries.

The value of our current simulation studies lies in providing a fresh conceptual framework that bridges two disparate experimental observations—the long-range effects of low Kr expression and the under-scaling of Bcd in the posterior—through the well-documented and modelled regulatory properties of the gap gene network to explain the emergence of size-dependent features in the posterior. Our simulation results show that a reduced Kr level can on its own exert long-range and dynamic impacts on gap expression properties (in terms of both expression level and boundary position) in the posterior. The simulated long-range impact of Kr level reduction is reminiscent of the long-range effects on *eve* patterning observed in *Kr* and *Kr*/*+*, but not *kni*, mutant embryos^61^. A gene circuit model for *Kr* mutant embryos^50^ inferred that the anterior shift of the Gt boundaries was caused by a lack of Kr repression; our simulations suggest that a ∼1.6 dosage of the *Kr* gene has similar effects, albeit of modest size. It is notable that the gene circuit model exhibits the Kr-mediated effects of size-dependent Bcd alteration without any additional training. Additional training with expression data from the size-selected lines would probably change the genetic interaction matrix, a zygotic property. Our simulations therefore suggest that the patterning differences originate maternally and do not require any divergence of the zygotic gap genes between the two lines. A related final point concerns the ability of the gene circuit model to both canalize high Bcd variation^21^,^50^ and exhibit posterior under-scaling as reported here. These apparently contradictory behaviours may be reconciled by noting that the perturbations are qualitatively different. The simulations in Manu *et al.* correspond to random Bcd variation in nominally isogenic embryos, whereas the perturbation modelled here is a systematic difference stemming from a large change in embryo length between two genetically distinct lines^21^. That the same gene circuit underlies both types of the simulated—and empirically observed—behaviours highlights the complexity and plasticity of gene regulatory network dynamics under different perturbations.

An important suggestion of our current study is that the *Kr* mRNA level divergence in relation to size can stem from the scaling properties of the Bcd gradient at the location of the *Kr* expression domain. Thus the AP patterning behaviour in the posterior part of the embryo with respect to scaling could be viewed/explained as being controlled primarily by the dynamic operation of the gap gene network, where the system's initial state is determined by the Bcd input and mediated by *Kr*. The role of Bcd as a positive input for *Kr* expression is well documented both experimentally and *in silico*^19^,^25^,^48^,^49^,^50^, and our current simulation results further support this relationship. Importantly, our study suggests that this relationship has a role in mediating the long-range and dynamic effects on gap gene expression to guide the dynamic emergence of size-dependent features in the posterior trunk region of the embryo. This supports the view that the entire AP axis is patterned as a single unified dynamic system in which the size-driven changes in maternal positional information are interpreted locally in individual nuclei, but in a globally coordinated manner. It remains to be elucidated whether our suggested Bcd-*Kr* relationship is the sole mediator for size-dependent patterning behaviour in the posterior at a quantitative level.

Our experimental design takes advantage of two inbred lines that were selected from wild populations for laying large and small eggs^39^. The fact that the spatial registry of gap expression boundaries and their temporal movements in these lines exhibit an overall similarity to one another and to other well-characterized wild-type strains suggests that neither of these lines is genetically ‘defective' with respect to AP patterning. To further evaluate whether our observed *Kr* expression level divergence is indeed a property that is associated with embryo size (as opposed to other genetic differences between the inbred lines used in this study), we analysed *Kr* expression level in relation to embryo length within individual lines separately. Our analysis reveals a significant (inverse) correlation in embryos from the large-egg line, though not in embryos from the small-egg line (Supplementary Fig. 10a,b). These results document that *Kr* level can diverge in embryos where size differences are not caused by genetic differences. These results also further support our suggestion that Bcd gradient properties associated with embryo size, that is, its scaling properties, can impact *Kr* expression level. We note that, for extracting scaling-specific features, within-line analyses are inherently less sensitive than between-line analyses due to the limited size variations within a line (Supplementary Fig. 10). We attribute our inability to detect a significant correlation between *Kr* level and embryo length in the small-egg line to this reduced experimental sensitivity. This also further underscores the value of the experimental strategy used in our current study.

Developmental robustness is an important concept that can guide how we view and interpret the relevant events that take place on different time scales. It has been proposed that within-species scaling properties of the Bcd gradient can be traced fundamentally to a dynamic relationship between *bcd* gene copy number expansion and nurse cell size expansion during oogenesis^6^. Under the framework of a recently proposed model of Tissue Expansion-Modulated Maternal Morphogen Scaling (TEM^3^S)^6^, this dynamic relationship also imposes constraints on Bcd gradient properties in relation to embryo size in different dipteran species during evolution. Relative to *D*. *melanogaster* embryos, the larger-sized *D*. *yakuba* embryos appear to exhibit both weakened gap expression levels (for *Kr* and posterior *kni* and *gt* domains) and under-scaled characteristics in the posterior^45^. This suggests a possibility that scaling-specific features and their mechanistic underpinnings within a species may also be applicable to evaluations of AP patterning properties across species. The early *Drosophila* embryo undergoes rapid nuclear divisions and morphological changes^24^,^41^,^62^,^63^. How cells (nuclei) in this dynamic environment decode positional information remains an open question^27^,^31^,^32^,^34^,^64^,^65^. The observed wave-like initiation and recovery of under-scaling in the posterior trunk region suggest that, to fully understand developmental robustness with respect to scaling, knowledge of the process of achieving the final outcome (that is, phenotype) is as important as that of the outcome itself. In addition, the temporal constraints on molecular processes imposed by the rapid morphological progression of the embryo may also be relevant to events and changes that take place on the evolutionary time scale.

## Methods

### Embryo collection and RNA FISH

The two inbred lines (#2.46.4 and #9.17.1) used in this study were gifts of Drs Cecelia Miles and Martin Kreitman and they had been selected from wild populations for laying large or small eggs^39^. Flies were reared at 25 °C and 0–4-h embryos were collected from 5–10-day-old females. For convenience, we refer to the embryos collected from the two inbred lines as large and small embryos, respectively. For mRNA FISH, digoxigenin (dig)-labelled RNA probes were synthesized *in vitro* using PCR products or plasmid (for *hb*) as templates as before^66^. Gene-specific PCR primers are listed in Supplementary Table 2. The primers for *kni* (forward: 5′-CGGAATTCAGTCCTTCTTTGGCCGCTCTTAC-3′; reverse: 5′-TAATACGACTCACTATAGGGTCTAGATTAGACACACACGAATATTC-3′) and the plasmid for synthesizing *hb* probes (CZ4001) were as described^66^. In our mRNA FISH analysis (refs 33, 66), the hybridization step was carried out for 40 h at 60 °C. After hybridization with dig-labelled RNA probes, embryos were incubated overnight at 4 °C with sheep anti-dig primary antibody (Roche, 1:400), followed by a 1-hour incubation at room temperature with goat anti-sheep secondary antibody conjugated with Alexa Fluor 594 (Life technologies, 1:400). DNA counterstain with 4,6-diamidino-2-phenylindole (DAPI) (Sigma, 1:1,250) was performed for 10 min at room temperature before mounting using the Prolong Gold medium (Invitrogen). Coverslip ‘bridges' were used to minimize physical distortions and the mounted embryos on slides were let to equilibrate for at least 10 h at 4 °C before imaging.

### Image acquisition

To allow comparisons of the expression profiles of a gene between embryos from the two inbred lines, we ensured that embryo collection, mRNA FISH and imaging were all performed on a side-by-side basis. During imaging, selection criteria were used to ensure that an embryo to be imaged: (1) was at the stage of interest (nc13 or nc14 before gastrulation); (2) was judged to be laterally oriented with the anterior and posterior poles aligned horizontally (that is, without obvious tilt along the AP axis); and (3) did not exhibit gross deformations due to experimental manipulations or morphological defects. Image acquisition was performed on Zeiss Imager Z1 ApoTome microscope in conjunction with the AxioVision 4.8 software. All images were captured on the mid-sagittal plane of the embryo under linear settings without any normalizations or other nonlinear adjustments^31^. To avoid pixel intensity saturation, we first selected a stained embryo that was judged to have the highest fluorescence signals (according to software determination) for calibrations. In particular, we used this embryo to set an exposure time such that the highest intensity pixels remained unsaturated, and this chosen exposure time, together with all the other microscopic and software settings, was fixed throughout an entire imaging cycle for all images. Therefore, absolute intensity levels for each gene could be compared between embryos from the two inbred lines, but not between genes irrespective of line. For each embryo, two images were taken, the first of which captured the fluorescent signals (× 10 objective, capturing the entire embryo at its mid-sagittal section), followed by that for the DIC channel (× 20 objective, focused on the dorsal side for quantifying the progression of membrane invagination). All images (in TIF format) had 1382 × 1040 resolution and 8-bit pixel depth.

### Time class classification

The nuclear cycle (nc13 or nc14) of an embryo was first determined by evaluating the number of nuclei on the dorsal side^63^. For embryos at nc14 before gastrulation (∼60 min into the interphase), we divided them into nine time classes based on nuclear morphology and membrane invagination. Nuclear morphology provides a sensitive measure of time for embryos at early times of nc14 (refs 63, 67), whereas membrane invagination is a reliable measure of later time^20^,^29^,^68^,^69^. In our analysis, the nuclear length of an embryo was obtained by averaging measurements of five nuclei on the dorsal side captured by the DAPI channel. Membrane invagination ratio was calculated as a percentage of invagination depth relative to cortex length. We made measurements at three different locations on the dorsal side of an embryo and used the average for ranking embryos. Distance measurements for both nuclear length and membrane invagination depth were conducted using ImageJ.

Embryos that had <15% membrane invagination ratio on the dorsal side, estimated not to have exceeded ∼25 min into the interphase^67^, were grouped into the early time classes T1 and T2, whose division was based on the simple ranking of the measured nuclear lengths. Embryos that had >15% membrane invagination ratio were divided into the remainder of the time classes (T3–T9) according to the simple ranking of the percentage of membrane imagination at arbitrary cut-offs (25, 35, 45, 55, 65 and 75%). The chosen method led to both a fine temporal resolution and a sufficient number of embryos at individual time classes for each of the two inbred lines and for each of the genes analysed (see Supplementary Table 1). We estimated that the time classes of T3–T5 represent mid-nc14 embryos (∼25–40 min into nc14) and those of T6–T9 represent late nc14 embryos (∼40–60 min into nc14)^69^. The nine time classes have an approximate time of: 0–13.5, 13.5–25, 25–31, 31–35, 35–38, 38–42, 42–45, 45–48 and 48–60 min after the thirteenth nuclear division, respectively (see Fig. 6 for further details).

### Image processing to obtain gene expression profiles

Image processing was performed using Matlab (MathWorks) as follows^31^. Briefly, an embryo was first oriented along its AP axis with the anterior end to the left and dorsal side up using the software with manual supervision when applicable. In our analysis, the length of an embryo was first recorded and divided into 50 equal-sized bins along the AP axis; this allows the extraction of a bin-location-based intensity profile on the dorsal side of the embryo such that the extracted profile has effectively been ‘projected' onto the AP axis and expressed as a function of relative AP position irrespective of embryo size or shape. Specifically, the mRNA FISH intensities from the image of the embryo were extracted by moving a scanning circle (of 61 pixels) along the dorsal side of an embryo immediately below (basal to) the nuclear layer as detected by DAPI signals. Background signals on the DAPI channel were also used for defining the embryo bounds with manual assistance whenever necessary. Intensity values for each scanning location were expressed, in arbitrary units, as the mean of all pixels within the scanning circle and an embryo's raw intensity profile smoothed by applying a Gaussian filter. Fluorescent signals at non-expression regions were then used in an embryo-specific background subtraction across all positions, without any further adjustments unless otherwise specified.

### Boundary position and movement and domain width

To determine the boundary position of a gap expression domain, the mean of three highest intensity values of a domain was set as 1 for normalizing intensity values at all positions for this domain. Boundary position was defined as the relative AP position (*ξ*=*x*/*L*) at which the normalized intensity value is 0.5 (see Fig. 4a). For the posterior *tll* domain, we avoided the most posterior three bins for normalization and boundary calculation to prevent capturing erroneous intensities from pole cells. We also avoided boundary calculations whenever prohibited because of low mRNA abundance that rendered such measurements unreliable. In particular, the boundaries of *gt*1 and *gt*2 become detectable only after T4, whereas the mRNA level of the anterior *hb* expression domain decreases so rapidly during early nc14 that the anterior boundary (*hb*1) no longer exists after T4 (Supplementary Fig. 1). Our analysis also did not include mRNA expression domains that never reached an appreciable level suitable for reliable boundary measurements (such as the posterior *Kr* domain). Thus the data shown in Fig. 1c,d and Supplementary Fig. 2 do not have boundaries for certain genes at certain times.

The width of an expression domain was defined (see Fig. 4a) as the distance between its anterior and posterior boundaries (both expressed as relative AP positions). Specifically, for each time class, we first measured the domain width of an expression domain in individual embryos to obtain a mean (±s.d.) of the large or small embryos as a group, 〈*W*_large_〉 and 〈*W*_small_〉, respectively. We then calculated the width difference at each time class as Δ*W*=〈*W*_large_〉*−*〈*W*_small_〉. The s.d. of Δ*W* was estimated by 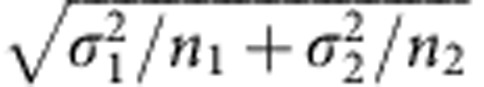, where *n*_1_ and *n*_2_ are sample numbers for the two groups of embryos, and *σ*_1_ and *σ*_2_ are their respective s.d. The *gt* ant2 domain is composed of two sub-domains upon its split at later nc14. For the posterior *tll* domain, the domain width is defined as the distance between the *tll*3 boundary and the posterior end. When calculating boundary movements as a function of time, s.d. of the difference (distance) of the means was also estimated by 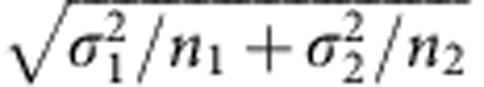.

### Scaling coefficient and threshold-crossing analysis

To analyse the scaling property of an expression boundary, we pooled the large and small embryos at a given time class to generate a scatter plot of boundary position (*ξ*) against normalized embryo length *L*/〈*L*〉, where 〈*L*〉 is the mean of the two inbred lines' mean embryo lengths in this study. The slope of the fitted linear regression in such a scatter plot is defined as *S* (see Supplementary Fig. 3). Threshold-crossing analysis was performed using the interpolated mean (unnormalized) expression profiles of a group of embryos to obtain positions at arbitrary thresholds. Data analyses and figure generation were performed using Matlab (MathWorks) or Microsoft Excel. Unless otherwise specified, all values shown are mean±s.d. and all *P* values are from two-tailed Student's *t*-test.

### Simulations

The simulations were performed using the gene circuit model of Manu *et al.*^19^,^47^. The model simulates the gene regulation of Hb, Kr, Gt and Kni in nuclei lying between 0.35 and 0.92 EL on the AP axis of the embryo. The simulation begins after the twelfth nuclear division and lasts until gastrulation, a duration of ∼71 min (ref. 62). The state variables are the concentrations of each gap protein in each nucleus, 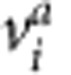, where *a* is the gap gene and *i* is the nucleus. The concentrations evolve according to the coupled differential equations


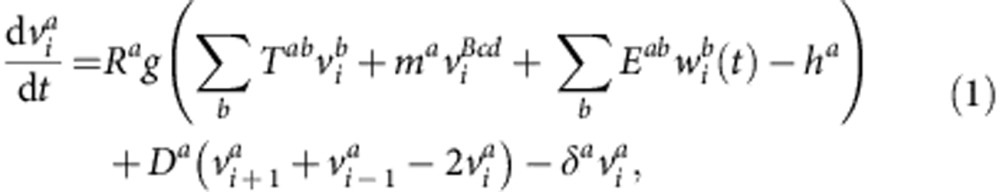


where the first term represents synthesis, the second represents the diffusion of gap proteins, and the third represents first-order degradation. *R*^*a*^, *D*^*a*^ and *δ*^*a*^ are the maximum synthesis rate, diffusion coefficient, and degradation coefficient, respectively. In the synthesis term 
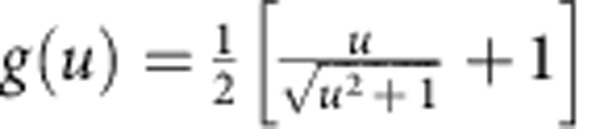
 is a sigmoidal thresholding function. *T* is the genetic interconnectivity matrix between the gap genes, in which positive and negative elements signify activation and repression, respectively. 
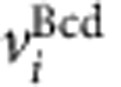
 is the concentration of Bcd and *m*^*a*^ is the strength of regulation by Bcd. The model also includes the time-dependent effects of upstream regulators Cad and Tll, where 
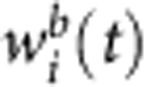
 is the concentration and *E*^*ab*^ is the strength of regulation. *h*^*a*^ is a threshold term. The initial conditions for the gap genes were from nc12 data in the FlyEx database (http://urchin.spbcas.ru/flyex/)^61^,^70^. Kr, Gt and Kni protein is first detected in nc13 (ref. 29), and hence their initial concentrations were zero. Cad and Tll data were from^21^. Except as noted below, we utilized the model reported in Manu *et al.*^19^ (model 007) without modification, and refer the readers to that paper for the details of numerical simulation and parameter inference. The parameter values are listed in Supplementary Table 3.

We designate the unmodified model, which utilizes a Bcd profile *v*^Bcd^(*x*)=*A* exp(−*x*/*λ*) with *A*=351 (in a.u.) and *λ*=0.1651 EL, as the control simulation. For the simulation of reduced synthesis rates (Supplementary Fig. 8), one of *R*^*Hb*^, *R*^*Kr*^, *R*^*Gt*^, *R*^*Kni*^ or all together were reduced by 20%. For the simulations of Bcd scaling in larger embryos (Fig. 6 and Supplementary Fig. 9), we considered two effects. First, the length scale of Bcd does not change with embryo length^46^, a fact we represent by reducing the relative-unit length scale of Bcd by 20%—the approximate ratio of embryo lengths of large and small embryos—to 0.1321 EL in the model. Second, larger embryos were observed to have more *bcd* mRNA and higher protein expression at the anterior^46^. This was represented in the model by increasing the value of *A*. The simultaneous reduction of relative length scale and increase in *A* causes the scaled Bcd to intersect with the control Bcd at a critical position^6^,^46^. We investigated the dependence of simulated gap gene expression patterns on the location of the critical position by varying *A* (Supplementary Fig. 9). Larger values of *A* shift the critical position toward the posterior and vice versa.

We measured the boundary positions and peak expression level in domains by fitting a smoothed cubic spline to the model output using the CSAPS function of MATLAB (MathWorks Inc.). Boundary positions were calculated as the position where the simulated expression level is at the mean of the consecutive extrema.

## Additional information

**How to cite this article:** Wu, H. *et al.* Temporal and spatial dynamics of scaling-specific features of a gene regulatory network in *Drosophila*. *Nat. Commun.* 6:10031 doi: 10.1038/ncomms10031 (2015).

## Supplementary Material

Supplementary Information Supplementary Figures 1-10, Supplementary Tables 1-3 and Supplementary References.

## References

[b1] Waddington C. H. Canalization of development and the inheritance of aquired characters. Nature 150, 563–565 (1942).

[b2] Patel N. H. & Lall S. Precision patterning. Nature 415, 748–749 (2002). 11845195 10.1038/415748a

[b3] Umulis D. M. & Othmer H. G. Mechanisms of scaling in pattern formation. Development 140, 4830–4843 (2013). 24301464 10.1242/dev.100511 PMC3848184

[b4] Lander A. D. Pattern, growth, and control. Cell 144, 955–969 (2011). 21414486 10.1016/j.cell.2011.03.009 PMC3128888

[b5] Cheung D., Miles C., Kreitman M. & Ma J. Adaptation of the length scale and amplitude of the Bicoid gradient profile to achieve robust patterning in abnormally large *Drosophila melanogaster* embryos. Development 141, 124–135 (2014). 24284208 10.1242/dev.098640 PMC3865754

[b6] He F. *et al.* Fundamental origins and limits for scaling a maternal morphogen gradient. Nat. Commun. 6, 6679 (2015). 25809405 10.1038/ncomms7679 PMC4375784

[b7] Crickmore M. A. & Mann R. S. The control of size in animals: insights from selector genes. Bioessays 30, 843–853 (2008). 18693263 10.1002/bies.20806 PMC2656436

[b8] Ben-Zvi D., Shilo B. Z. & Barkai N. Scaling of morphogen gradients. Curr. Opin Genet. Dev. 21, 704–710 (2011). 21873045 10.1016/j.gde.2011.07.011

[b9] Wartlick O., Mumcu P., Julicher F. & Gonzalez-Gaitan M. Understanding morphogenetic growth control – lessons from flies. Nat. Rev. Mol. Cell Biol. 12, 594–604 (2011). 21850035 10.1038/nrm3169

[b10] Rogers K. W. & Schier A. F. Morphogen gradients: from generation to interpretation. Annu. Rev. Cell Dev. Biol. 27, 377–407 (2011). 21801015 10.1146/annurev-cellbio-092910-154148

[b11] Wartlick O. *et al.* Dynamics of Dpp signaling and proliferation control. Science 331, 1154–1159 (2011). 21385708 10.1126/science.1200037

[b12] Hamaratoglu F., de Lachapelle A. M., Pyrowolakis G., Bergmann S. & Affolter M. Dpp signaling activity requires Pentagone to scale with tissue size in the growing *Drosophila* wing imaginal disc. PLoS Biol. 9, e1001182 (2011). 22039350 10.1371/journal.pbio.1001182 PMC3201923

[b13] Ben-Zvi D., Pyrowolakis G., Barkai N. & Shilo B. Z. Expansion-repression mechanism for scaling the Dpp activation gradient in *Drosophila* wing imaginal discs. Curr. Biol. 21, 1391–1396 (2011). 21835621 10.1016/j.cub.2011.07.015

[b14] Fried P. & Iber D. Dynamic scaling of morphogen gradients on growing domains. Nat. Commun. 5, 5077 (2014). 25295831 10.1038/ncomms6077

[b15] Kicheva A. *et al.* Coordination of progenitor specification and growth in mouse and chick spinal cord. Science 345, 1254927 (2014). 25258086 10.1126/science.1254927 PMC4228193

[b16] Spradling A. C. in The Development of Drosophila melanogaster eds Bates M., Martinez-Arias A. 1–70Cold Spring Harbor Press (1993).

[b17] Jaeger J., Manu & Reinitz J. Drosophila blastoderm patterning. Curr. Opin. Genet. Dev. 22, 533–541 (2012). 23290311 10.1016/j.gde.2012.10.005

[b18] Houchmandzadeh B., Wieschaus E. & Leibler S. Establishment of developmental precision and proportions in the early *Drosophila* embryo. Nature 415, 798–802 (2002). 11845210 10.1038/415798a

[b19] Manu *et al.* Canalization of gene expression in the *Drosophila* blastoderm by gap gene cross regulation. PLoS Biol. 7, e1000049 (2009). 19750121 10.1371/journal.pbio.1000049 PMC2653557

[b20] Dubuis J. O., Samanta R. & Gregor T. Accurate measurements of dynamics and reproducibility in small genetic networks. Mol. Syst. Biol. 9, 639 (2013). 23340845 10.1038/msb.2012.72 PMC3564256

[b21] Porcher A. & Dostatni N. The bicoid morphogen system. Curr. Biol. 20, R249–R254 (2010). 20219179 10.1016/j.cub.2010.01.026

[b22] Lohr U., Chung H. R., Beller M. & Jackle H. Bicoid–morphogen function revisited. Fly 4, 236–240 (2010). 20404518 10.4161/fly.4.3.11862 PMC3322503

[b23] Chen H., Xu Z., Mei C., Yu D. & Small S. A system of repressor gradients spatially organizes the boundaries of Bicoid-dependent target genes. Cell 149, 618–629 (2012). 22541432 10.1016/j.cell.2012.03.018 PMC3535481

[b24] Gebelein B. & Ma J. Regulation in the early *Drosophila* embryo. Rev. Cell Biol. Mol. Med. (in press) (2015).

[b25] Jaeger J. *et al.* Dynamic control of positional information in the early *Drosophila* embryo. Nature 430, 368–371 (2004). 15254541 10.1038/nature02678

[b26] Bieler J., Pozzorini C. & Naef F. Whole-embryo modeling of early segmentation in *Drosophila* identifies robust and fragile expression domains. Biophys. J. 101, 287–296 (2011). 21767480 10.1016/j.bpj.2011.05.060 PMC3136765

[b27] Bergmann S. *et al.* Pre-steady-state decoding of the Bicoid morphogen gradient. PLoS Biol. 5, e46 (2007). 17298180 10.1371/journal.pbio.0050046 PMC1790957

[b28] Fowlkes C. C. *et al.* A quantitative spatiotemporal atlas of gene expression in the *Drosophila* blastoderm. Cell 133, 364–374 (2008). 18423206 10.1016/j.cell.2008.01.053

[b29] Surkova S. *et al.* Characterization of the *Drosophila* segment determination morphome. Dev. Biol. 313, 844–862 (2008). 18067886 10.1016/j.ydbio.2007.10.037 PMC2254320

[b30] Crombach A., Wotton K. R., Cicin-Sain D., Ashyraliyev M. & Jaeger J. Efficient reverse-engineering of a developmental gene regulatory network. PLoS Comput. Biol. 8, e1002589 (2012). 22807664 10.1371/journal.pcbi.1002589 PMC3395622

[b31] He F. *et al.* Probing intrinsic properties of a robust morphogen gradient in *Drosophila*. Dev. Cell 15, 558–567 (2008). 18854140 10.1016/j.devcel.2008.09.004 PMC2629455

[b32] Gregor T., Tank D. W., Wieschaus E. F. & Bialek W. Probing the limits to positional information. Cell 130, 153–164 (2007). 17632062 10.1016/j.cell.2007.05.025 PMC2253670

[b33] He F., Ren J., Wang W. & Ma J. Evaluating the *Drosophila* Bicoid morphogen gradient system through dissecting the noise in transcriptional bursts. Bioinformatics 28, 970–975 (2012). 22302571 10.1093/bioinformatics/bts068 PMC3315720

[b34] Tostevin F., ten Wolde P. R. & Howard M. Fundamental limits to position determination by concentration gradients. PLoS Comput. Biol. 3, e78 (2007). 17465676 10.1371/journal.pcbi.0030078 PMC1857820

[b35] He F. *et al.* Shaping a morphogen gradient for positional precision. Biophys. J. 99, 697–707 (2010). 20682246 10.1016/j.bpj.2010.04.073 PMC2913175

[b36] Cheung D. & Ma J. Probing the impact of temperature on molecular events in a developmental system. Sci. Rep. 5, 13124 (2015). 26286011 10.1038/srep13124 PMC4541335

[b37] Kuntz S. G. & Eisen M. B. *Drosophila* embryogenesis scales uniformly across temperature in developmentally diverse species. PLoS Genet. 10, e1004293 (2014). 24762628 10.1371/journal.pgen.1004293 PMC3998915

[b38] Lott S. E., Kreitman M., Palsson A., Alekseeva E. & Ludwig M. Z. Canalization of segmentation and its evolution in *Drosophila*. Proc. Natl Acad. Sci. USA 104, 10926–10931 (2007). 17569783 10.1073/pnas.0701359104 PMC1891814

[b39] Miles C. M. *et al.* Artificial selection on egg size perturbs early pattern formation in *Drosophila melanogaster*. Evolution 65, 33–42 (2011). 20636356 10.1111/j.1558-5646.2010.01088.x PMC2988983

[b40] Driever W. & Nüsslein-Volhard C. The bicoid protein determines position in the *Drosophila* embryo in a concentration dependent manner. Cell 54, 95–104 (1988). 3383245 10.1016/0092-8674(88)90183-3

[b41] Liu J. & Ma J. Dampened regulates the activating potency of Bicoid and the embryonic patterning outcome in *Drosophila*. Nat. Commun. 4, 2968 (2013). 24336107 10.1038/ncomms3968 PMC3902774

[b42] Conrad B. & Antonarakis S. E. Gene duplication: a drive for phenotypic diversity and cause of human disease. Annu. Rev. Genomics Hum. Genet. 8, 17–35 (2007). 17386002 10.1146/annurev.genom.8.021307.110233

[b43] Semina E. V. *et al.* Cloning and characterization of a novel bicoid-related homeobox transcription factor gene, RIEG, involved in Rieger syndrome. Nat. Genet. 14, 392–399 (1996). 8944018 10.1038/ng1296-392

[b44] de Lachapelle A. M. & Bergmann S. Precision and scaling in morphogen gradient read-out. Mol. Syst. Biol. 6, 351 (2010). 20212523 10.1038/msb.2010.7 PMC2858443

[b45] Fowlkes C. C. *et al.* A conserved developmental patterning network produces quantitatively different output in multiple species of *Drosophila*. PLoS Genet. 7, e1002346 (2011). 22046143 10.1371/journal.pgen.1002346 PMC3203197

[b46] Cheung D., Miles C., Kreitman M. & Ma J. Scaling of the Bicoid morphogen gradient by a volume-dependent production rate. Development 138, 2741–2749 (2011). 21613328 10.1242/dev.064402 PMC3109599

[b47] Manu *et al.* Canalization of gene expression and domain shifts in the *Drosophila* blastoderm by dynamical attractors. PLoS Comput. Biol. 5, e1000303 (2009). 19282965 10.1371/journal.pcbi.1000303 PMC2646127

[b48] Hoch M., Schroder C., Seifert E. & Jackle H. *Cis*-acting control elements for Kruppel expression in the *Drosophila* embryo. EMBO J. 9, 2587–2595 (1990). 2114978 10.1002/j.1460-2075.1990.tb07440.x PMC552291

[b49] Hoch M., Seifert E. & Jackle H. Gene expression mediated by cis-acting sequences of the Kruppel gene in response to the *Drosophila* morphogens bicoid and hunchback. EMBO J. 10, 2267–2278 (1991). 2065664 10.1002/j.1460-2075.1991.tb07763.x PMC452917

[b50] Kozlov K., Surkova S., Myasnikova E., Reinitz J. & Samsonova M. Modeling of gap gene expression in *Drosophila* Kruppel mutants. PLoS Comput. Biol. 8, e1002635 (2012). 22927803 10.1371/journal.pcbi.1002635 PMC3426564

[b51] Kim Y. *et al.* Context-dependent transcriptional interpretation of mitogen activated protein kinase signaling in the *Drosophila* embryo. Chaos 23, 025105 (2013). 23822503 10.1063/1.4808157 PMC3689791

[b52] Lohr U., Chung H. R., Beller M. & Jackle H. Antagonistic action of Bicoid and the repressor Capicua determines the spatial limits of *Drosophila* head gene expression domains. Proc. Natl Acad. Sci. USA 106, 21659–21700 (2009). 10.1073/pnas.0910225106 PMC2788481 19959668

[b53] Krotov D., Dubuis J. O., Gregor T. & Bialek W. Morphogenesis at criticality. Proc. Natl Acad. Sci. USA 111, 3683–3688 (2014). 24516161 10.1073/pnas.1324186111 PMC3956198

[b54] Papatsenko D. & Levine M. The *Drosophila* gap gene network is composed of two parallel toggle switches. PloS ONE 6, e21145 (2011). 21747931 10.1371/journal.pone.0021145 PMC3128594

[b55] Vakulenko S., Manu, Reinitz J. & Radulescu O. Size regulation in the segmentation of *Drosophila*: interacting interfaces between localized domains of gene expression ensure robust spatial patterning. Phys. Rev. Lett. 103, 168102 (2009). 19911861 10.1103/PhysRevLett.103.168102 PMC2977926

[b56] Struhl G., Johnston P. & Lawrence P. A. Control of *Drosophila* body pattern by the hunchback morphogen gradient. Cell 69, 237–249 (1992). 1568245 10.1016/0092-8674(92)90405-2

[b57] Hülskamp M., Pfeifle C. & Tautz D. A morphogenetic gradient of hunchback protein organizes the expression of the gap genes Krüppel and Knirps in the early *Drosophila* embryo. Nature 346, 577–580 (1990). 2377231 10.1038/346577a0

[b58] Schulz C. & Tautz D. Autonomous concentration-dependent activation and repression of Kruppel by hunchback in the *Drosophila* embryo. Development 120, 3043–3049 (1994). 7607091 10.1242/dev.120.10.3043

[b59] Holloway D. M. & Spirov A. V. Mid-embryo patterning and precision in *Drosophila* segmentation: Kruppel dual regulation of hunchback. PloS ONE 10, e0118450 (2015). 25793381 10.1371/journal.pone.0118450 PMC4368514

[b60] Papatsenko D. & Levine M. S. Dual regulation by the Hunchback gradient in the *Drosophila* embryo. Proc. Natl Acad. Sci. USA 105, 2901–2906 (2008). 18287046 10.1073/pnas.0711941105 PMC2268557

[b61] Surkova S. *et al.* Quantitative dynamics and increased variability of segmentation gene expression in the *Drosophila* Kruppel and knirps mutants. Dev. Biol. 376, 99–112 (2013). 23333947 10.1016/j.ydbio.2013.01.008

[b62] Foe V. E. & Alberts B. M. Studies of nuclear and cytoplasmic behaviour during the five mitotic cycles that precede gastrulation in *Drosophila* embryogenesis. J. Cell Sci. 61, 31–70 (1983). 6411748 10.1242/jcs.61.1.31

[b63] Liu J. & Ma J. Uncovering a dynamic feature of the transcriptional regulatory network for anterior-posterior patterning in the *Drosophila* embryo. PloS ONE 8, e62641 (2013). 23646132 10.1371/journal.pone.0062641 PMC3639989

[b64] He F. & Ma J. A Spatial point pattern analysis in *Drosophila* blastoderm embryos evaluating the potential inheritance of transcriptional states. PloS ONE 8, e60876 (2013). 23593336 10.1371/journal.pone.0060876 PMC3621909

[b65] Porcher A. *et al.* The time to measure positional information: maternal hunchback is required for the synchrony of the Bicoid transcriptional response at the onset of zygotic transcription. Development 137, 2795–2804 (2010). 20663819 10.1242/dev.051300

[b66] Liu J. & Ma J. Fates-shifted is an F-box protein that targets Bicoid for degradation and regulates developmental fate determination in *Drosophila* embryos. Nat. Cell Biol. 13, 22–29 (2011). 21170036 10.1038/ncb2141 PMC3074934

[b67] Fung J. C., Marshall W. F., Dernburg A., Agard D. A. & Sedat J. W. Homologous chromosome pairing in *Drosophila melanogaster* proceeds through multiple independent initiations. J. Cell Biol. 141, 5–20 (1998). 9531544 10.1083/jcb.141.1.5 PMC2132734

[b68] Merrill P. T., Sweeton D. & Wieschaus E. Requirements for autosomal gene activity during precellular stages of *Drosophila melanogaster*. Development 104, 495–509 (1988). 3151484 10.1242/dev.104.3.495

[b69] Royou A., Field C., Sisson J. C., Sullivan W. & Karess R. Reassessing the role and dynamics of nonmuscle myosin II during furrow formation in early *Drosophila* embryos. Mol. Biol. Cell 15, 838–850 (2004). 14657248 10.1091/mbc.E03-06-0440 PMC329397

[b70] Pisarev A., Poustelnikova E., Samsonova M. & Reinitz J. FlyEx, the quantitative atlas on segmentation gene expression at cellular resolution. Nucleic Acids Res. 37, D560–D566 (2009). 18953041 10.1093/nar/gkn717 PMC2686593

