# Supplementary material for: Temporal and spatial dynamics of scaling-specific features of a gene regulatory network in Drosophila
Source: Nat Commun. 2015 Dec 8;6:10031. doi: 10.1038/ncomms10031 (PMC4686680; doi:10.1038/ncomms10031)
Supplement: Supplementary Information — Supplementary Figures 1-10, Supplementary Tables 1-3 and Supplementary References. [file ncomms10031-s1.pdf]

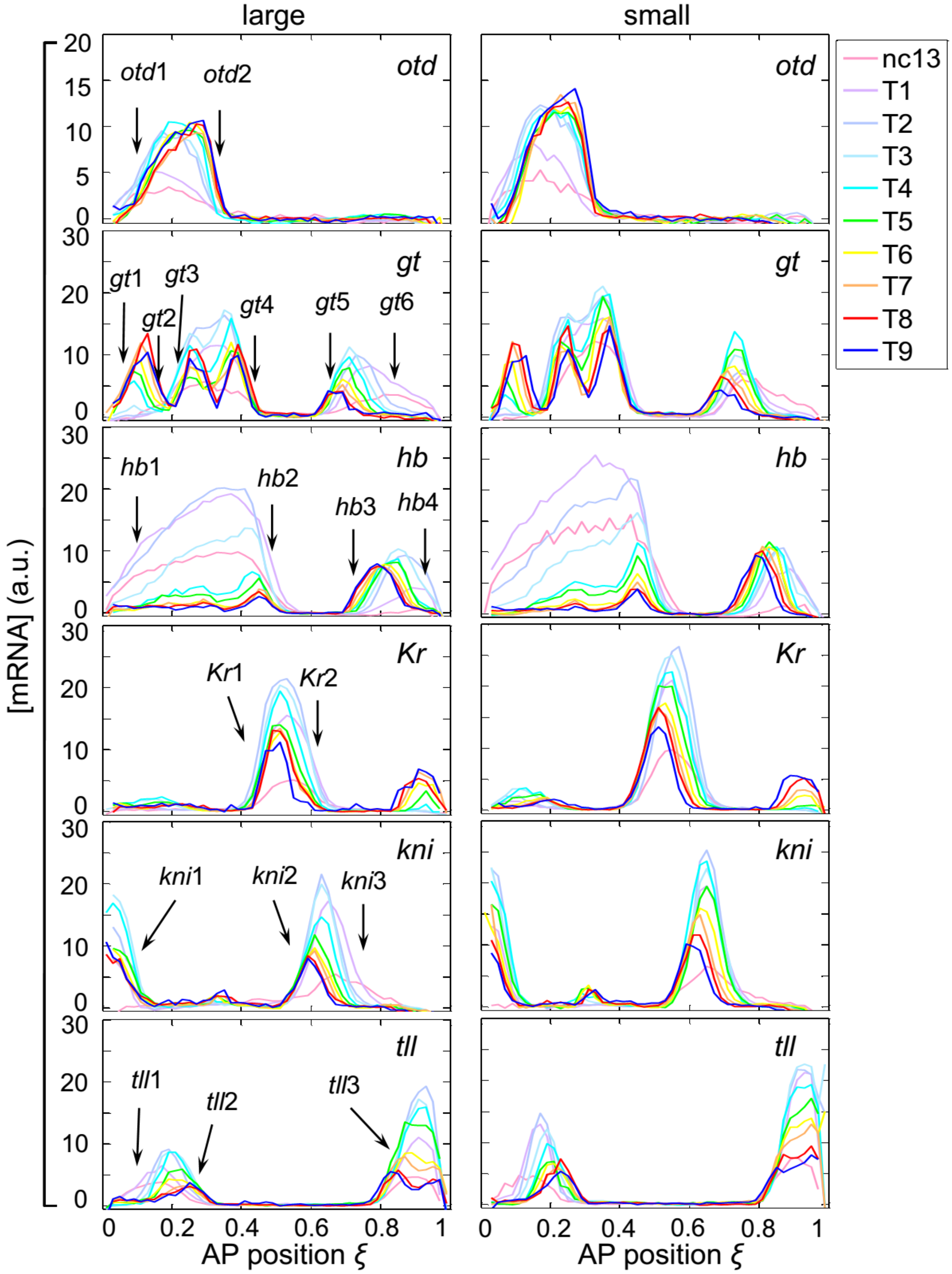

Supplementary Fig. 1. Expression profiles of gap genes in large and small embryos. Shown are the mean mRNA expression profiles (as a function of fractional embryo length $\xi = x/L$) of the indicated six gap genes (*otd*, *gt*, *hb*, *Kr*, *kni* and *tll*) at each of the ten time classes in large (left panels) and small (right panels) embryos. mRNA concentrations are in arbitrary units (a.u). All profiles are unadjusted except an embryo-specific subtraction of background intensities. On the left panel, each of the 20 expression boundaries analyzed in the study is indicated with a name given.

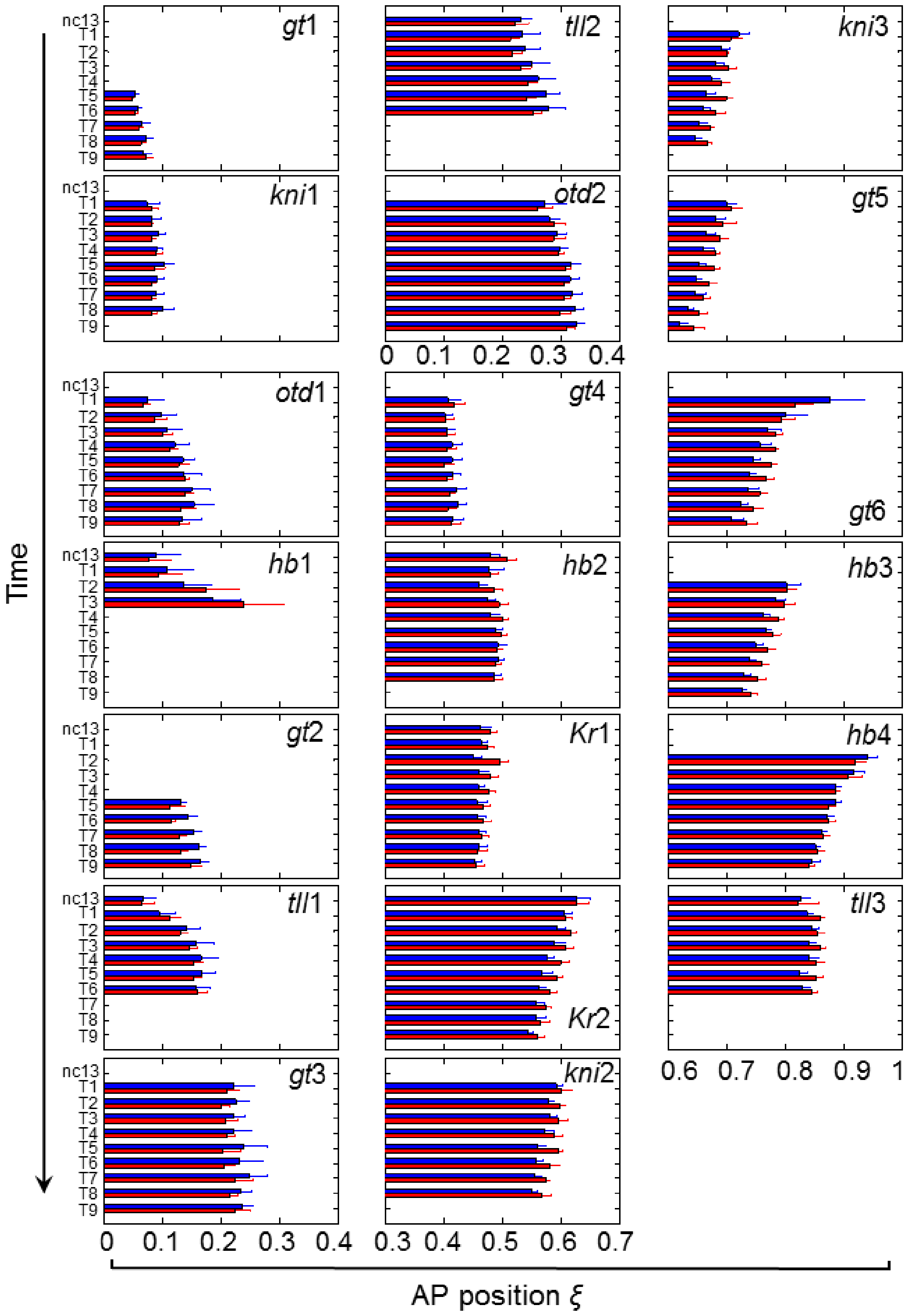

Supplementary Fig. 2. Boundary position dynamics of gap gene expression in large and small embryos. Shown are the measured mean positions (and s.d.) of each of the 20 expression boundaries in large (blue) and small (red) embryos at each of the ten time classes. Panels for individual boundaries are arranged according to their AP positions (with top left most anterior and bottom right most posterior). Examples discussed in text: At the onset of nc14 interphase, the *Kr2* boundary position is similar (0.606 ± 0.015 and 0.607 ± 0.012 at T1 for large and small embryos, respectively; P = 0.74; Student's t test). At mid nc14, it becomes more anteriorly positioned in large embryos (e.g., 0.577 ± 0.010 and 0.600 ± 0.015 at T4 for large and small embryos, respectively; $P = 8.8\times10^{-5}$), a different that persists (e.g., 0.542 ± 0.010 and 0.560 ± 0.010 at T9 for large and small embryos, respectively; $P = 3.5\times10^{-4}$). Moving span example: *gt6* moves a distance of 0.170 ± 0.015 (from 0.877 ± 0.060 at T1 to 0.707 ± 0.023 at T9) in large embryos, and 0.086±0.009 (from 0.819 ± 0.030 at T1 to 0.733 ± 0.020 at T9) in small embryos.

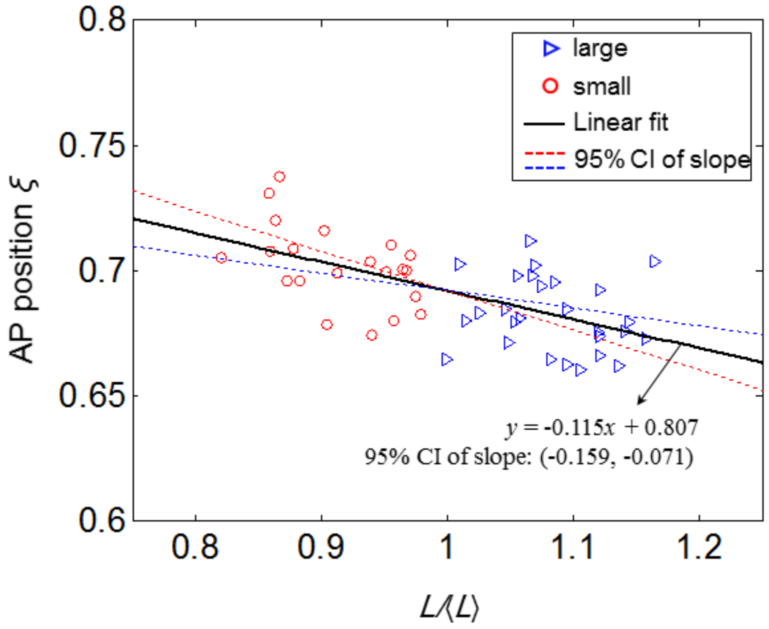

Supplementary Fig. 3. Definition of scaling coefficient *S*. Shown is an illustration of the calculation of scaling coefficient. Here the *kni*3 boundary in a pooled group of large and small embryos at time class T3 is used as an example. In this figure, the boundary position in individual embryos (expressed as relative AP position ξ) is plotted against normalized embryo length $L/\langle L\rangle$, where $\langle L\rangle$ is the mean of the two lines' mean lengths in the current study. In the plot shown, the slope of the linear regression line is defined as scaling coefficient *S*. The bounds of the 95% CI of the fitted slope are also shown.

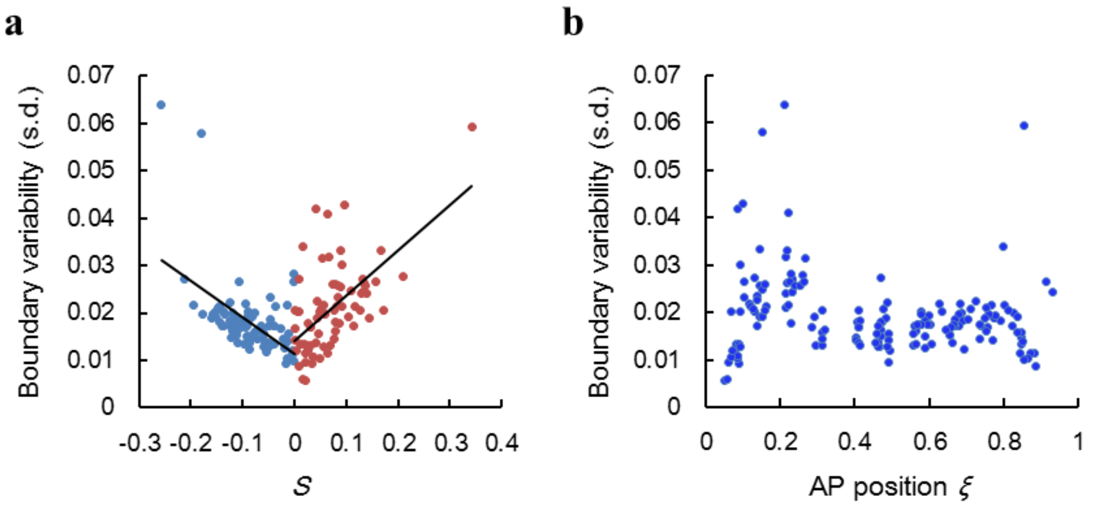

Supplementary Fig. 4. Relationships between boundary precision and scaling coefficient or boundary position. (a) Shown is a scatter plot of boundary variability in pooled large and small embryos against the calculated scaling coefficient values. This is a compilation of all boundaries at all times, with the datapoints split at $S = 0$ for linear fitting on the two sides separately. The results shown in this panel support the conclusion that scaling contributes to the overall precision of gap expression boundaries. (b) Shown is a scatter plot of boundary variability against relative AP position ξ. In comparison with panel a, the results shown in this panel exhibit less discernible trends, except that the variations of expression boundaries near middle portions of the embryo are more "uniform" than those toward either poles.

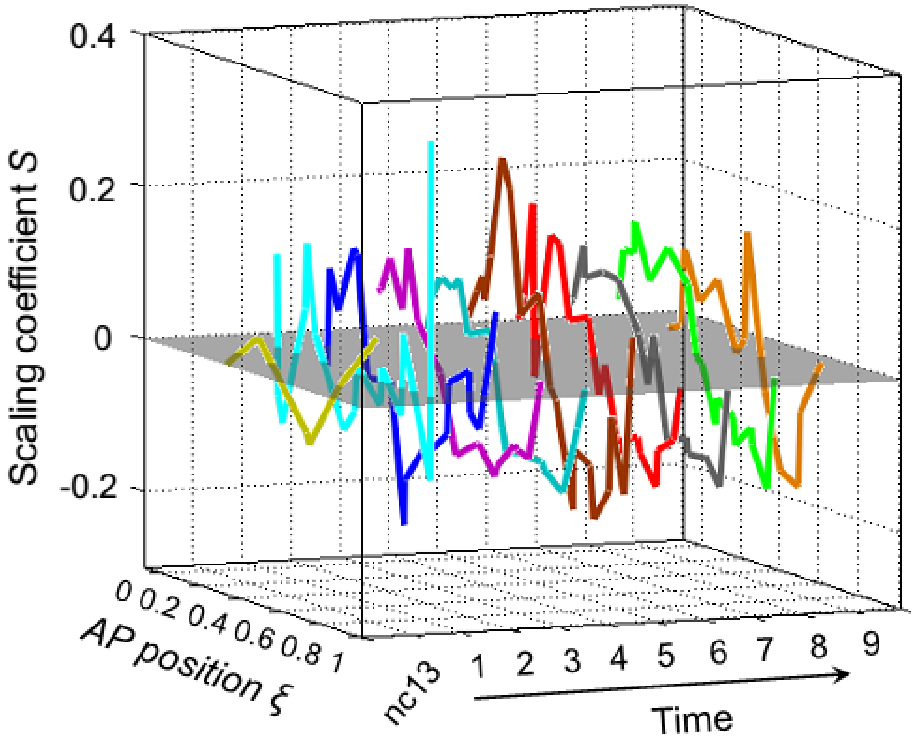

Supplementary Fig. 5. Time evolution of the scaling landscape along the AP axis. Shown is a 3D representation of scaling coefficient S as a function of AP position ξ and as a function of time. This figure is a compilation of all the measured S values. For the purpose of visual clarity, this figure does not show CI data; see Fig. 3 for 95% CI shown as shaded bands. A gray plane denotes $S = 0$.

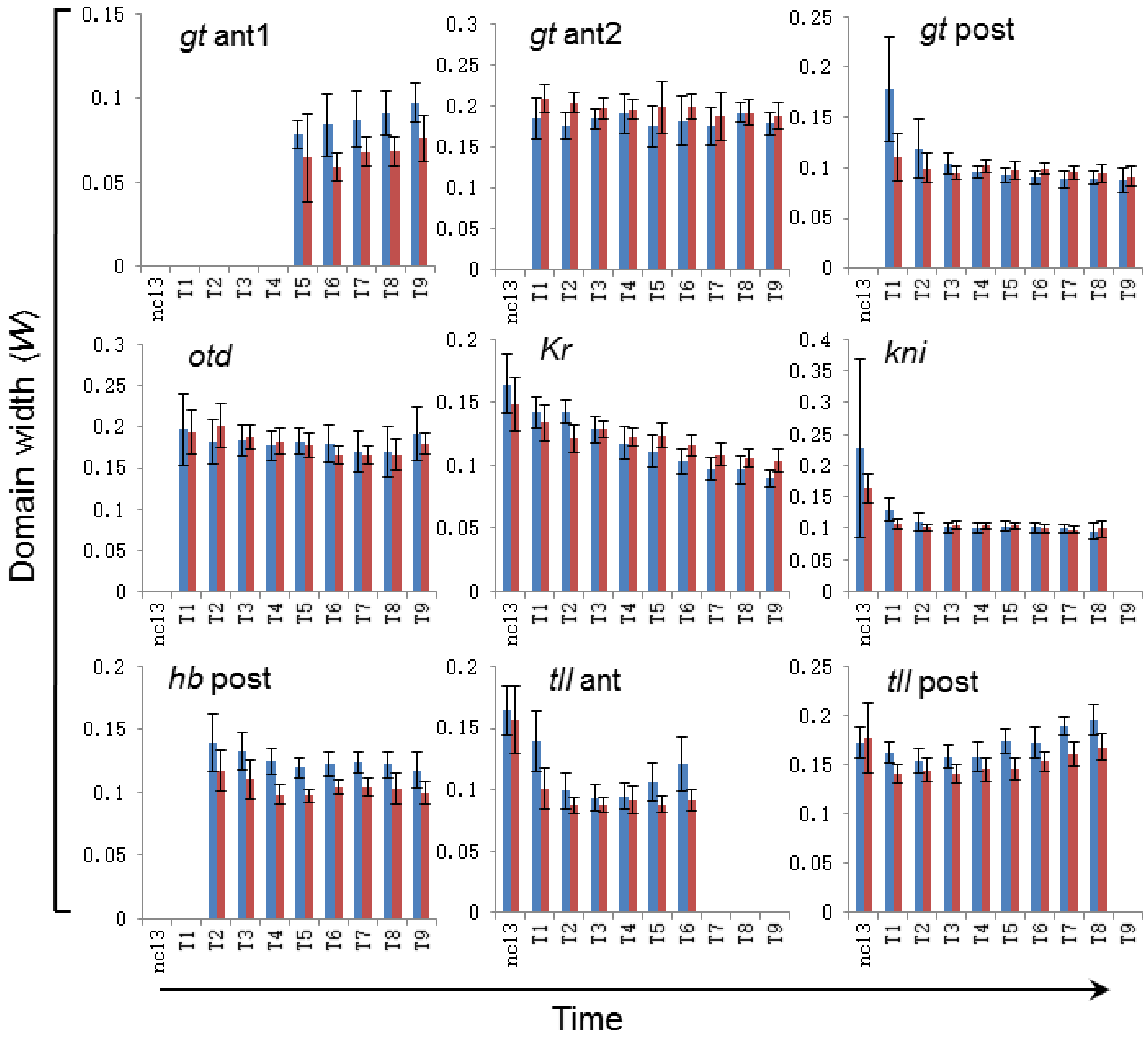

Supplementary Fig. 6. Gap gene expression domain widths. Shown are the mean widths (with s.d.) of individual gap expression domains at different time classes for large (light blue) and small (dark red) embryos. Domain widths are expressed in values relative to embryo length. Each domain shown is defined by two boundaries as listed below: *gt* ant1 (*gt*1 and *gt*2), *gt* ant2 (*gt*3 and *gt*4), *gt* post (*gt*5 and *gt*6), *otd* (*otd*1 and *otd*2), *Kr* (*Kr*1 and *Kr*2), *kni* (*kni*2 and *kni*3), *hb* post (*hb*3 and *hb*4), *tll* ant (*tll*1 and *tll*2), *tll* post (*tll*3 and the posterior end). Ant, anterior; post, posterior.

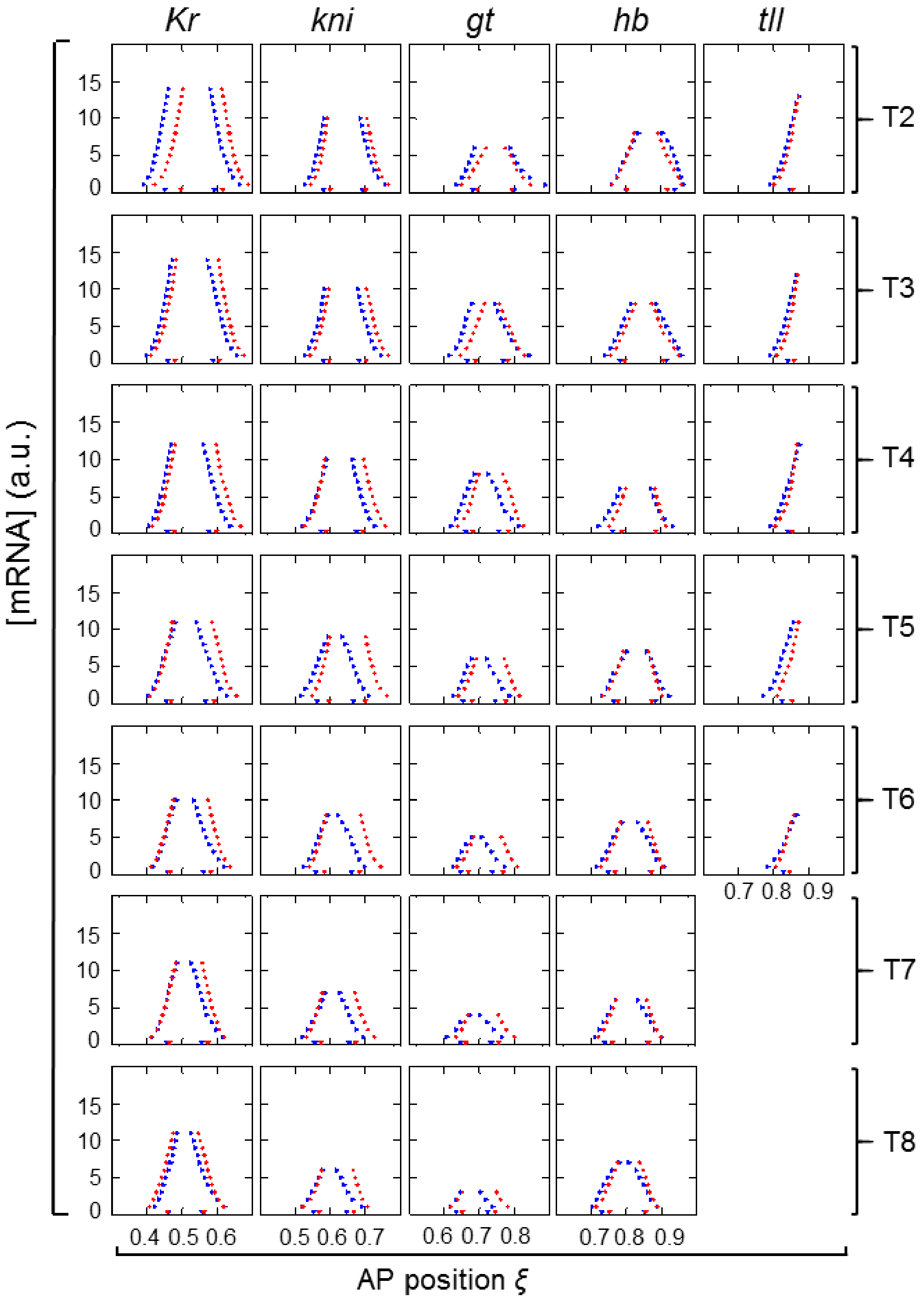

Supplementary Fig. 7. Threshold-crossing positions of gap expression profiles. Shown are relative AP positions at which a mean profile of gap gene expression crosses given thresholds in large (blue) and small (red) embryos at the indicated times. Measured boundary positions are shown at the bottom of each panel for reference.

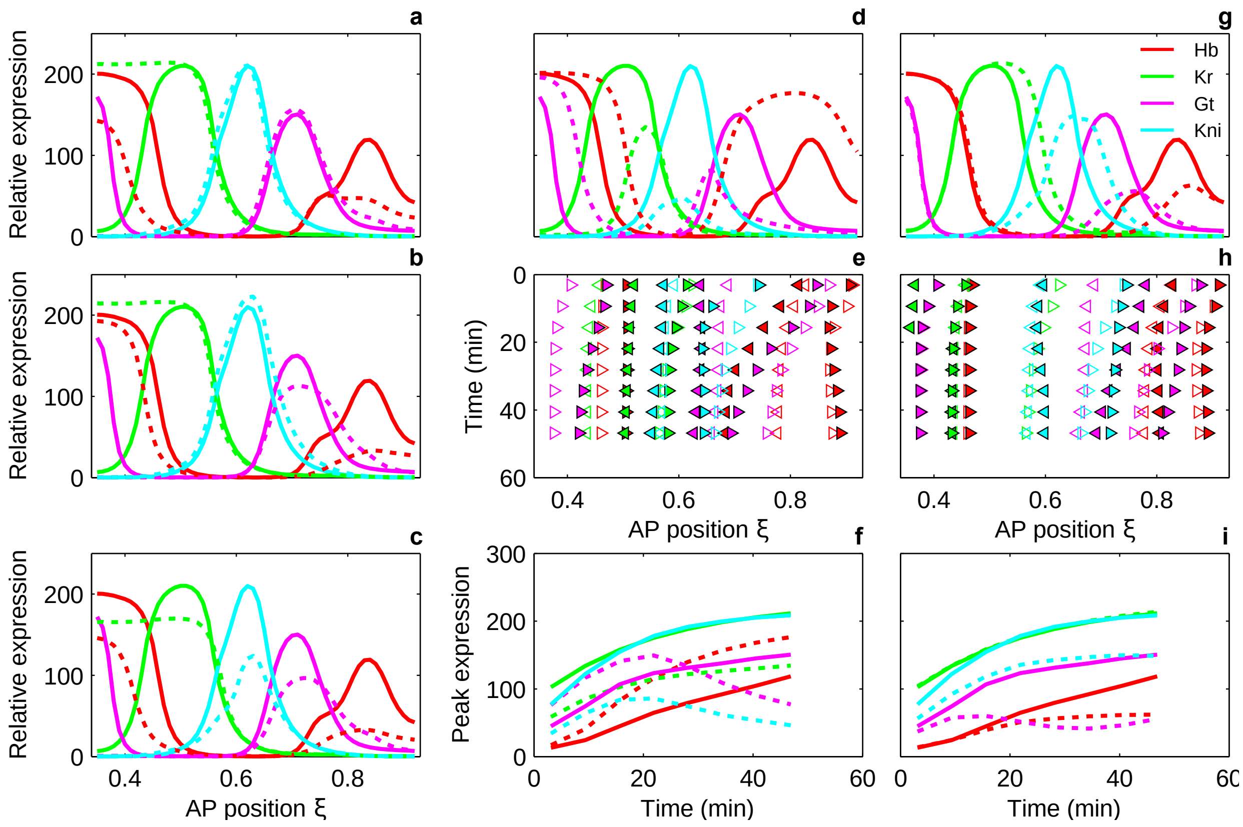

Supplementary Fig. 8. Simulation of reduced synthesis rates in the gene circuit model of the gap genes. (a-d, g) Gap protein expression patterns at 46.875 min (T8) in simulations with reduced rates of maximum synthesis. Control and reduced synthesis rate simulations are plotted with solid and dashed lines, respectively. The color key is shown in panel g. The maximum rate of synthesis was reduced by 20% for Hb (a), Gt (b), Kr (d), Kni (g), or all of the gap proteins together (c). (e,h) The time course of boundary positions in simulations with reduced Kr (e) or Kni (h) synthesis rates. See Fig. 6 legend for time points. Anterior and posterior boundaries are represented by left- and right-pointing triangles, respectively. Control and reduced synthesis rate simulations are shown as open and filled triangles, respectively. (f,i) Time series of the expression level at the peak of the posterior Hb, central Kr, posterior Gt, and abdominal Kni domains, in simulations with reduced Kr (f) or Kni (i) synthesis rates. Reduction of Hb or Gt synthesis rates leads to lower expression levels for those proteins and local effects on boundary positions. Reduction of Kr synthesis rate causes reductions in the expression level of Kr, abdominal Kni, and posterior Gt domains, posterior shifts of boundaries lying anterior to the Kr peak and anterior shifts of boundaries lying to the posterior of the Kr peak. Reduction of Kni synthesis rate induces reduced central Kni, posterior Gt, and posterior Hb, but not central Kr domain. Additionally, it leads to posterior, instead of anterior, shifts of the domains posterior to the Kr peak.

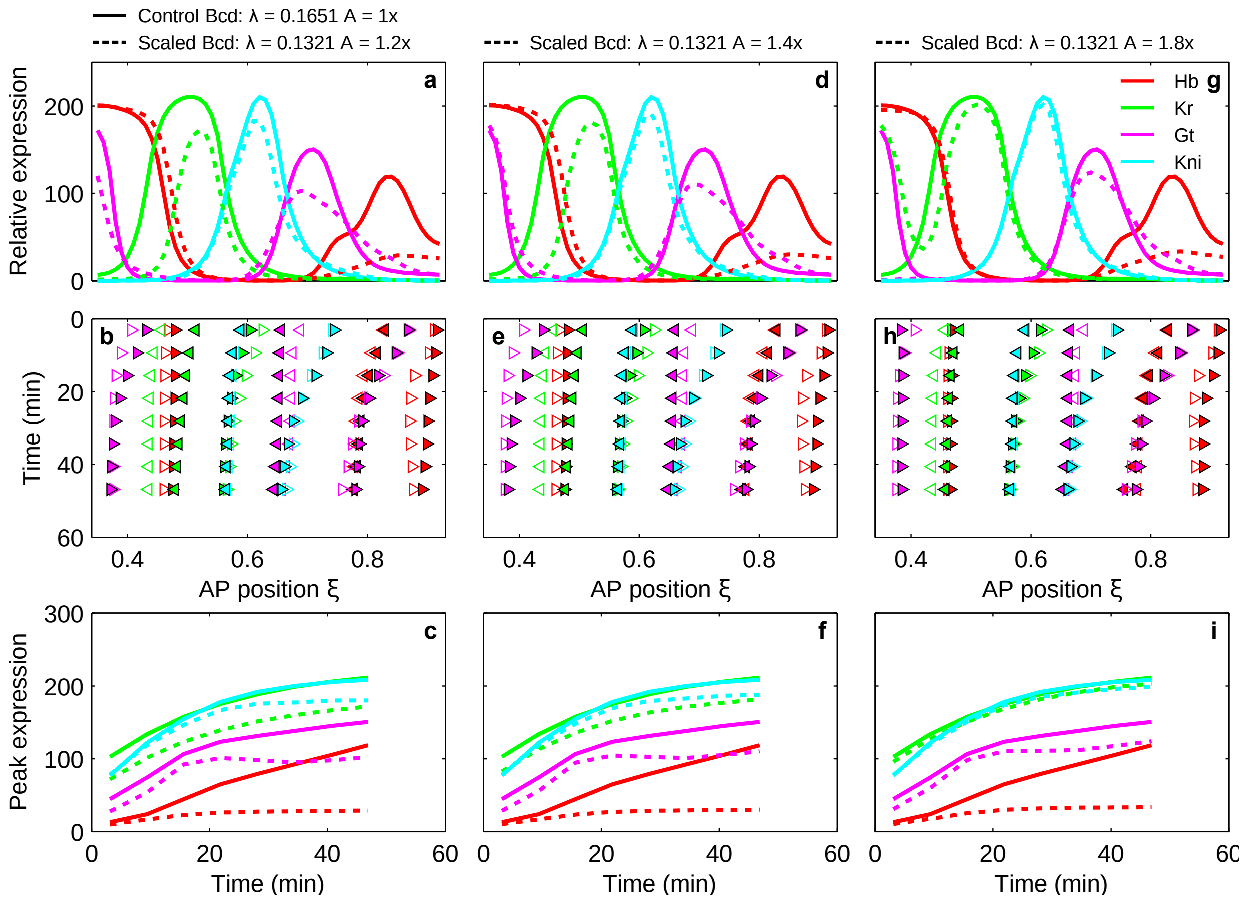

Supplementary Fig. 9. The effect of varying the critical position in simulations with the gene circuit model. (a,d,g) Gap protein expression patterns at 46.875 min (T8) in simulations with scaled Bcd. The parameters of the Bcd gradient profiles used in simulations are listed above the panels. Here the relative length scale of the control Bcd, 0.1651 EL, was reduced by 20% to 0.1321 EL in the scaled simulations. Control and scaled Bcd simulations are plotted with solid and dashed lines, respectively. The color key is shown in panel g. (b,e,h) The time course of boundary positions. Anterior and posterior boundaries are represented by left- and right-pointing triangles, respectively. Control and scaled Bcd simulations are shown as open and filled triangles, respectively. (c,f,i) Time series of the expression level at the peak of the posterior Hb, central Kr, posterior Gt, and abdominal Kni domains. (a-c) *A* is 1.2x control; critical position is $\xi = 0.121$. (d-f) *A* is 1.4x control; critical position is $\xi = 0.222$. (g-i) *A* is 1.8x control; critical position is $\xi = 0.389$. The critical position (where the two Bcd gradients intersect) moves to the posterior as *A* is increased (see Method). Therefore, the simulations with *A*=1.2x of control have the largest deficit of Bcd concentration in the central Kr domain. The anterior shifts of Kr2, Kni2, Kni3, and Gt5 boundaries decrease as the critical position shifts to the posterior. In all simulations, the reduction in Kr expression at the domain peak is relatively constant in time, whereas the difference between control and scaled Bcd simulations for Gt and Kni increases in time. Shifting the critical position closer to the Kr domain reduces the magnitude of change in peak expression of Kr, Gt, and Kni.

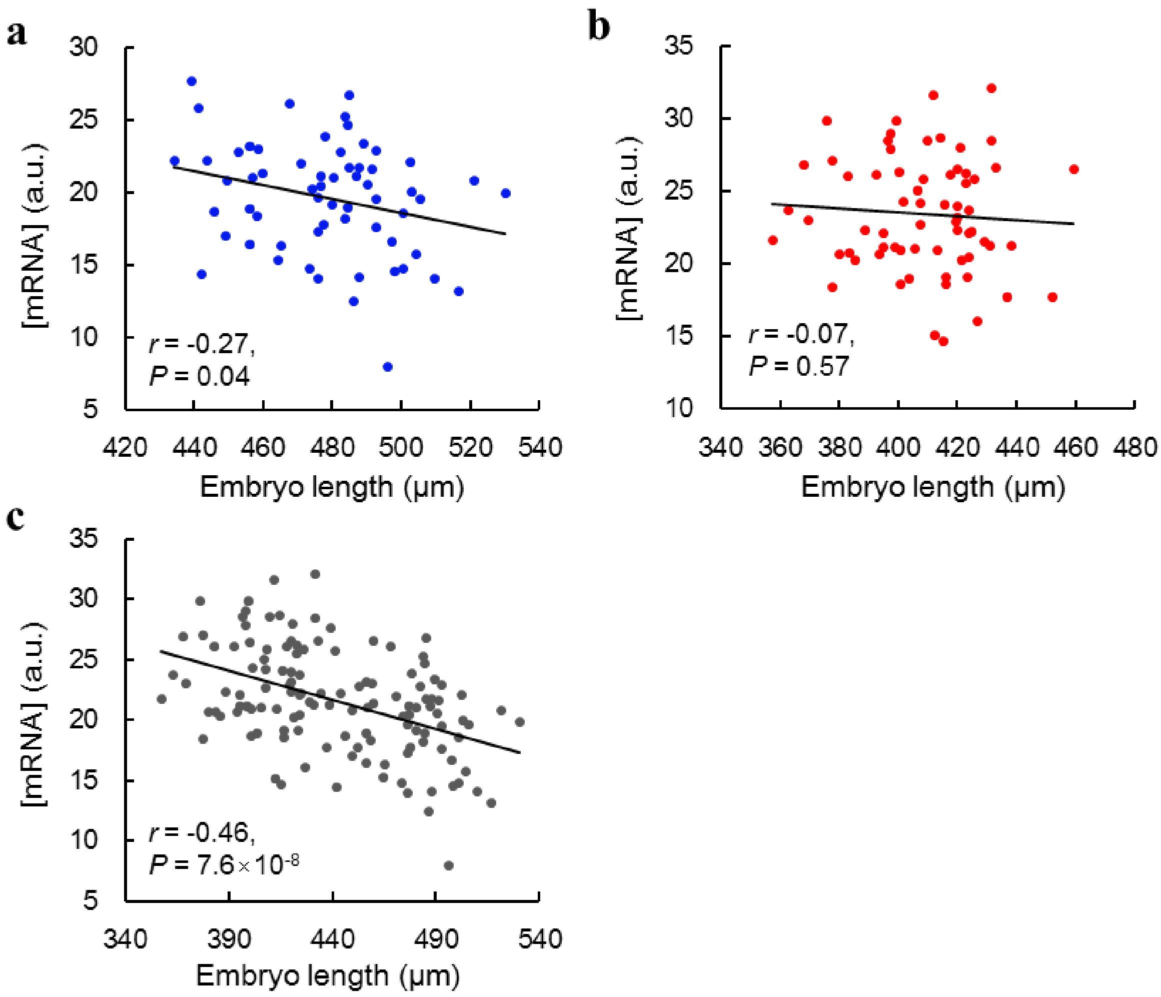

Supplementary Fig. 10. ***Kr*** **expression level in relation to embryo length.** Shown are scatter plots of *Kr* expression levels against embryo lengths for the large-egg line (a), small-egg line (b) or two lines pooled (c). In each panel, a linear regression line, Pearson correlation coefficient r and P value (from Pearson coefficient calculation) are also given. Data shown are from embryos at time classes T2 to T4, when *Kr* mRNA expression is around its peak. The results show that, while the inverse correlation detected in embryos from the large-egg line or the two lines pooled is significant, no significant correlation is detected in embryos from the small-egg line.

Supplementary Table 1: Embryo numbers for the indicated time classes and gap genes analyzed

Time class		nc13	T1	T2	T3	T4	T5	T6	T7	T8	T9
gt	large	21	21	24	22	14	11	18	9	13	10
	small	6	15	16	29	10	10	15	17	14	27
hb	large	27	13	30	39	17	13	30	19	22	13
	small	11	12	25	35	17	12	21	28	18	20
kni	large	7	7	8	27	14	8	18	17	16	11
	small	6	11	6	21	9	9	14	16	11	14
Kr	large	9	18	13	35	13	15	20	26	13	7
	small	11	14	9	40	17	14	15	22	29	36
otd	large	7	23	17	45	19	23	21	20	24	22
	small	9	11	12	39	16	9	12	21	16	21
tll	large	19	16	38	35	24	17	21	28	27	19
	small	6	14	13	38	22	12	27	23	14	32

Supplementary Table 2: PCR primer sequences

Gene	Forward primer	Reverse primer
otd	5'-GGACTAGTGGTGTGGTTCAAGAATC<br>GCCG-3'	5'-CCCTCGAGTAATACGACTCACTATA<br>GGGCAGGCTGTTCTGGGAGAAGG-3'
gt	5'-GGACTAGTCTGATGCACCACCACCA<br>GTAC-3'	5'-CCCTCGAGTAATACGACTCACTATA<br>GGGAGAGGAGTGGACCTTTAGGCG-3'
Kr	5'-GGACTAGTCCAACTTCTGGCTGCAA<br>ACC-3'	5'-CCCTCGAGTAATACGACTCACTATA<br>GGGTCACTGGGTACGTGAGGGAT-3'
tll	5'-GGACTAGTGTCGCATTCTATACCAT<br>GTGCC-3'	5'-CCCTCGAGTAATACGACTCACTATA<br>GGGTGAGGCGCACA ATGGTGATG-3'

Note: T7 promoter sequence is underlined.

Supplementary Table 3: Parameter values of the gene circuit model used in this study. The parameter values are the same as model 007 of Manu *et al*. [1]. The degradation coefficients are reported as half lives below ($t_{1/2}^a = \ln 2 / \delta^a$).

**parameter**	**value**
R^{hb}	15.000
R^{Kr}	10.354
R^{gt}	15.000
R^{kni}	15.000
$T^{hb \leftarrow hb}$	0.021
$T^{hb \leftarrow Kr}$	-0.001
$T^{hb \leftarrow gt}$	0.022
$T^{hb \leftarrow kni}$	-0.112
$T^{Kr \leftarrow hb}$	-0.026
$T^{Kr \leftarrow Kr}$	0.035
$T^{Kr \leftarrow gt}$	-0.042
$T^{Kr \leftarrow kni}$	-0.062
$T^{gt \leftarrow hb}$	-0.028
$T^{gt \leftarrow Kr}$	-0.202
$T^{gt \leftarrow gt}$	0.007
$T^{gt \leftarrow kni}$	0.003
$T^{kni \leftarrow hb}$	-0.082
$T^{kni \leftarrow Kr}$	-0.000
$T^{kni \leftarrow gt}$	-0.017
$T^{kni \leftarrow kni}$	0.013

$T^{hb \leftarrow cad}$	0.004
$T^{hb \leftarrow tll}$	0.003
$T^{Kr \leftarrow cad}$	0.021
$T^{Kr \leftarrow tll}$	-0.203
$T^{gt \leftarrow cad}$	0.023
$T^{gt \leftarrow tll}$	-0.011
$T^{kni \leftarrow cad}$	0.020
$T^{kni \leftarrow tll}$	-0.189
m^{hb}	0.025
m^{Kr}	0.118
m^{gt}	0.256
m^{kni}	0.012
D^{hb}	0.166
D^{Kr}	0.200
D^{gt}	0.103
D^{kni}	0.200
$t_{1/2}^{hb}$	9.529
$t_{1/2}^{Kr}$	15.908
$t_{1/2}^{gt}$	9.438
$t_{1/2}^{kni}$	13.062

Supplementary References

1 Manu *et al*. Canalization of gene expression in the Drosophila blastoderm by gap gene cross regulation. *PLoS biology* **7**, e1000049, doi:10.1371/journal.pbio.1000049 (2009).
